# Age 18–30 Trajectories of Beverage‐Specific Binge Drinking Prevalence Over 30 Years for Men and Women in the United States

**DOI:** 10.1111/acer.70420

**Published:** 2026-10-15

**Authors:** Justin Jager, Jin Yan, Paul Espinoza, Megan E. Patrick, Katherine M. Keyes

**Affiliations:** ^1^ Department of Psychology University of Michigan Ann Arbor Michigan USA; ^2^ Survey Research Center, Institute for Social Research University of Michigan Ann Arbor Michigan USA; ^3^ T. Denny Sanford School of Social and Family Dynamics Arizona State University Tempe Arizona USA; ^4^ Department of Epidemiology Columbia University Mailman School of Public Health New York New York USA

**Keywords:** beverage type, binge drinking, historical trends, trajectories, young adulthood

## Abstract

**Background:**

Binge drinking contributes to substantial mortality, morbidity, and economic costs in the United States. Although trends in and determinants of overall binge drinking are well characterized, little is known about how binge drinking trends vary by beverage type (beer, wine, liquor) across young adulthood and over historical time. Understanding beverage‐specific patterns may clarify shifts in alcohol‐related risks and inform beverage‐specific policy.

**Methods:**

Data were drawn from the Monitoring the Future longitudinal study, which sampled representative cohorts of US high‐school seniors from 1976 through 2008 and followed them across seven waves through age 30 from 1988 through 2020 (*N* = 20,684). Piecewise latent‐growth models assessed age trends, cohort trends, and age‐by‐cohort interactions in overall, beer, wine, and liquor binge drinking prevalence separately for each sex.

**Results:**

For both sexes, overall binge drinking followed an inverted U pattern across age (increases from age 18–22, decreases through age 30); beer and liquor binge drinking followed the overall inverted U pattern, whereas wine was flat. Cohort trends varied sharply by age and differentially so by beverage type. For both sexes, historical decreases in overall binge drinking at the outset of young adulthood (age 18) were a function of historical decreases in beer and wine. However, due to historical increases in liquor and wine during the latter portion of young adulthood, for both sexes age 18 decreases in overall binge drinking were not maintained through age 30, where levels were flat for men and increased for women.

**Conclusions:**

Beverage‐specific trends reveal that historical declines in binge drinking at younger ages of young adulthood are concentrated in beer and wine, whereas increases in liquor and wine at older ages offset these initial declines. Beverage‐specific policies with evidence‐based efficacy and cost effectiveness, such as increasing price and reducing availability, may help reduce emerging risks related to liquor and wine binge drinking.

## Graphical abstract

Using US historical‐longitudinal data, this study shows young adult binge drinking decreases historically at younger ages but not older ages of young adulthood. Beverage‐specific trends reveal that historical declines at younger ages are concentrated in beer and wine, whereas historical increases in liquor and wine at older ages offset these initial declines, underscoring the need for beverage‐specific prevention policies.

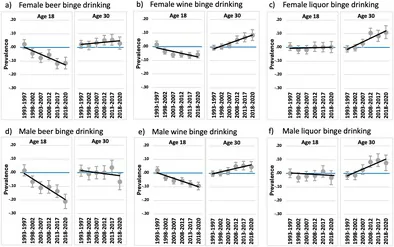

## 1 Introduction

Excessive alcohol use, including binge drinking (typically defined as 4+ drinks for women/5+ drinks for men; Centers for Disease Control and Prevention (CDC) 2012), not only plays a role in approximately 180,000 deaths annually within the United States (Esser 2024), it also is associated with a broad range of acute and chronic health conditions (Rehm et al. 2003; Shield et al. 2025), as well as economic costs of at least $250 billion annually within the United States (Sacks et al. 2015). Thus, excessive alcohol consumption, and binge drinking in particular, exerts a major burden on public health, the economy, and personal suffering. Understanding and preventing the effects of binge drinking remains an urgent and important challenge to public health.

Population level alcohol use is comprised of a mix of beverage types, such as beer, wine, and liquor. Historically, beer is the clear beverage of choice among men in terms of both alcohol volume and occasions of binge drinking (Dawson and Archer 1992; Lui et al. 2018; Stern et al. 2017); however, among women, wine is the most consumed in terms of volume (Dawson and Archer 1992; Lui et al. 2018), whereas occasions of binge drinking are highest for liquor (Stern et al. 2017). In addition to consumption patterns, drink characteristics also vary across beer, wine, and liquor (Gual et al. 1999; Kerr et al. 2005, 2008; Lemmens 1994), including liquid volume per drink (typically highest for beer, followed by wine and then liquor), ethanol volume per drink (typically highest for liquor, followed by wine and then beer), and across‐drink variability in ethanol volume (typically higher for liquor and wine than for beer). Available research also indicates differences across beer, wine, and liquor in the drivers of use. For example, expectations (e.g., degree of impairment, relaxation) and motivations for use vary across types of alcohol (Greenfield and Rogers 1999; Kuntsche et al. 2006; Pedersen et al. 2010). Finally, beer, wine, and liquor consumption are not only differentially taxed and marketed; they also are differentially responsive to taxation and marketing (Fogarty and Voon 2018; Subbaraman et al. 2020; Wilcox et al. 2015).

Given the pervasive differences in the patterns, drink characteristics, and drivers of beer, wine, and liquor consumption, research that disaggregates binge drinking by beer, wine, and liquor is an important complement to research focused on overall binge drinking. Nevertheless, compared to both age and historical trends in overall binge drinking, trends specific to beer, wine, and liquor binge drinking have received far less attention. Research documenting binge drinking trends specific to beer, wine, and liquor among young adults is particularly sparse. This relative lack of work focused on young adult patterns represents a potentially important knowledge gap, given that young adulthood is a formative period for later life alcohol consumption patterns and corresponding alcohol‐associated risks (Jackson et al. 2006; Patrick et al. 2024; Schulenberg et al. 2018; Tavolacci et al. 2019).

### 1.1 Age Trends and Historical Trends in Overall Young Adult Binge Drinking

Within the United States, binge drinking prevalence among young adults follows an inverted U‐shaped pattern characterized by increases across ages 18–22 and decreases across ages 22–30 (Chen et al. 2004; Jager et al. 2023; Lee and Sher 2018; Patrick et al. 2019). When aggregating across all young adults (i.e., combining those 18–30 into a singular group), historical analyses covering the last several decades indicate that binge drinking remained relatively stable (Grucza et al. 2018; Jager et al. 2023; Kerr et al. 2009); however, there is evidence of decreasing levels among recent (i.e., starting around 2021) cohorts of young adults (Patrick et al. 2025). Although across the past several decades binge drinking prevalence among young adults as an aggregate age group remained largely stable until recent declines, additional work (Jager et al. 2015, 2023; Patrick et al. 2019) indicates appreciable historical variation *within* the young adult age group. Specifically, at the outset of young adulthood (i.e., age 18) binge drinking has *decreased* (around 30% for women and 40% for men) across the last three decades. Yet this work also indicates that by the latter half of young adulthood (i.e., mid‐ to late‐20s) binge drinking has *increased* (around 50% for women and 10% for men) across the last three decades. Thus, relative to young adults of the past, today's young adults both enter young adulthood engaging in lower levels of binge drinking and exit young adulthood engaging in higher levels of binge drinking, with the degree of historical increases at the end of young adulthood being more pronounced for women. Given the across‐age reversal in historical trends for both sexes, potentially the timing of alcohol‐related harms among recent young adults has been shifting to older ages—particularly for women—within the 18–30 age band (Jager et al. 2023).

### 1.2 Age Trends and Historical Trends in Beverage‐Specific Young Adult Binge Drinking Frequency?

Whereas existing work focused on young adult trends in overall binge drinking provides important insights—namely, the ages within young adulthood the risks associated with binge drinking are particularly localized and how the location of those risks ebbs and flows across historical time—how these insights extrapolate to beer, wine, and liquor are not well understood. Regarding age trends, we were unable to locate existing research focused on age trends in beverage‐specific binge drinking across young adulthood. Related work (Kerr et al. 2004, 2013, 2024) focused on age trends in alcohol volume among all adults (i.e., 18+) indicates for both men and women that beer consumption evidences a steady linear decline across the adult lifecourse, whereas age trends in wine and liquor were comparatively flat. However, because of its focus on all adults, this work does not include sufficient coverage of young adulthood to delineate age trends within young adulthood. Additionally, because of its focus on alcohol volume, which does not necessarily change in parallel with binge drinking across age (Kerr et al. 2009), this work may not fully extrapolate to binge drinking.

Regarding beverage‐specific historical trends, available research focuses on annual per capita ethanol consumption (Fogarty and Voon 2018; Kerr et al. 2024; Martinez et al. 2019; Slater and Alpert 2025; Wilcox et al. 2015), national annual sales (Hart and Alston 2019), drinks per month (Kerr et al. 2004), or drinks per year (Kerr et al. 2013; Schiff et al. 2021) among all US adults. This work converges to indicate similar patterns for men and women characterized by consistent decreases in beer consumption over the last several decades along with more recent increases in wine and liquor over the last couple of decades, with the most recent work (Slater and Alpert 2025) indicating liquor overtaking beer in annual per capita ethanol consumption. However, due to its primary focus on general consumption of beer, wine, and liquor among all adults, how available research generalizes to historical trends in beer, wine, and liquor binge drinking among young adults as an aggregate group is not clear. One study (Stern et al. 2017) focused specifically on beverage‐specific historical trends in binge drinking among those ages 19 or 20 found no evidence of historical variation across 2002–2012; however, how these findings generalize to young adults of all ages and to a broader band of historical time is not clear.

Additionally, because no available work has examined whether historical trends in beer, wine, and liquor binge drinking vary within young adulthood, how the across‐age reversal in historical trends as well as the more pronounced across‐age reversal for women documented for overall binge drinking prevalence (Jager et al. 2015, 2023; Patrick et al. 2019) generalizes to beer, wine, and liquor is also unclear. That is, just as age trends and historical trends have been found to vary across beverage type, it could also be the case that the magnitude of the across‐age reversal varies by beverage type, which would indicate that some beverage types contribute to the reversal more than others. Moreover, these patterns could vary by sex, just as the magnitude of the across‐age reversal in historical variation of overall binge drinking varied by sex (Jager et al. 2015, 2023; Patrick et al. 2019).

### 1.3 Current Study

Using US national data that are both longitudinal and historical, we examine age and historical trends in young adult beverage‐specific binge drinking separately for men and women. We examine data on binge drinking beer, wine, and liquor from ages 18 to 30 among 33 successive cohorts who reached age 30 between 1988 and 2020. Specifically, we have three aims to delineate: (1) age trends (i.e., age main effect) within young adulthood by beverage type; (2) historical trends (i.e., cohort main effect) among young adults by beverage type; and (3) by beverage type whether historical trends among young adults vary by age (i.e., age‐by‐cohort interaction).

## 2 Methods

### 2.1 Respondents and Procedure

Monitoring the Future (MTF) includes an ongoing US national longitudinal study of substance use and other health behaviors among adolescents and adults (Miech et al. 2025; Patrick et al. 2025). Beginning in 1975, US samples of approximately 16,000 12th graders were drawn from about 135 public and private schools each year (Miech et al. 2025). Approximately 2450 individuals were randomly selected from each senior‐year cohort for follow‐up, with one random half being surveyed 1 year after high school and the other random half being surveyed 2 years after high school; each half was followed biennially through age 30 and every 5 years thereafter (Patrick et al. 2025). Respondents who reported drug use at baseline were oversampled for follow‐up; corrective weighting was used in the analyses. MTF uses a planned missingness design for data collection, where respondents are randomly assigned at baseline to one of 6 survey forms (i.e., questionnaire versions). All survey forms contain a set of core questions that are common across all forms. Each survey form also contains items that are not consistent across forms; measures of alcohol beverage type (i.e., beer, wine, and liquor) were only included on a single survey form (i.e., asked of 1/6th of all participants). The full sample was predominantly White (75.4%), evenly split by sex (50.9% female), and, on average, had parents who completed some college (for additional details, see Patrick et al. 2025). This study was approved by the IRB at University of Michigan.

In the present study, we focused on data for ages 18–30, which correspond to the first 7 waves of the MTF panel data. Respondents were 18 years old at Wave 1, 19–20 years old at Wave 2, 21–22 years old at Wave 3, 23–24 years old at Wave 4, 25–26 years old at Wave 5, 27–28 at Wave 6, and 29–30 at Wave 7. For the purposes of this study, analyses were limited to those respondents who were in 12th grade between 1976 and 2008 (i.e., those who had the opportunity to complete the age 29/30 surveys by 2020), who were randomly assigned the survey form that included the alcohol beverage type measures, and who provided data at one or more waves (*N* = 20,684). For the analytic sample, wave‐to‐wave retention rates between Waves 1 and 7 were 72.25%, 94.06%, 94.13%, 93.45%, 94.76%, and 93.87%, respectively. The retention rate between Waves 1 and 7 was 53.18%. These retention rates reflect the reality of long‐term longitudinal studies of drug use (Hansen et al. 1990; McCabe and West 2016) and compare reasonably well with other long‐term studies (Booker et al. 2011). Attrition analyses indicated that compared to those lost, those retained through Wave 7 were more likely to be female, to be white, to live in non‐urban areas, to report a higher high‐school GPA, to have more educated parents who are married, and to report lower senior‐year substance use, including binge drinking. These analyses also indicated that attrition rates were higher among recent cohorts, consistent with what is occurring with other longitudinal studies (Leeper 2019; Stedman et al. 2019). Although statistically significant, the effects of attrition were small in magnitude (i.e., *R* ^2^ = 0.03 for high‐school GPA and race/ethnicity, *R* ^2^ = 0.02 for sex, and *R* ^2^ < 0.005 for all other variables, including binge drinking; Jager et al. 2023). Thus, the amount of attrition in MTF is comparable to other prominent longitudinal studies, whereas the effects of attrition, including differential attrition across cohort, on sample estimates are minimal. Consistent with recent MTF analyses (Jager et al. 2024; Patrick 2025) we incorporated available analysis weights to adjust for both sampling and attrition (Patrick et al. 2023). These weights were developed to enable researchers to weight the MTF longitudinal panel data back to the initial nationally representative 12th grade MTF samples for relevant cohorts, accounting for the longitudinal panel sample selection process, including oversampling of those reporting substance use as well as panel attrition. Prior work indicates that these weights effectively adjust for the characteristics of participants who are lost to attrition and weight MTF longitudinal panel data back to the initial nationally representative sample of high‐school seniors even into midlife (Patrick et al. 2023).

### 2.2 Measures

#### 2.2.1 Binge Drinking Prevalence by Beverage Type

At each survey, frequency of binge drinking was measured for overall alcohol consumption as well as separately for beer, wine, and liquor. All measures used the same basic question format and response scale: “Think back over the last two weeks. How many times have you had five or more [*specific language varies by beverage specification]* in a row?”, where the specific language was: “…drinks…” for general alcohol consumption; “…12‐ounce cans of beer…” for beer; “…mixed drinks or shot glasses of hard liquor…” for liquor; and “…4‐ounce glasses of wine…” for wine. Response options included “none,” “once,” “twice,” “three to five times,” “six to nine times,” and “ten or more times.” For prevalence, overall, beer, wine, and liquor binge drinking were each coded as a dichotomous variable indicating any consumption of five or more drinks in a row during the last 2 weeks (yes, no). Because the specific combinations of beverages consumed during binge drinking occasions were not reported (i.e., only beverage‐specific drinking occasions that included 5+ drinks of the same beverage were reported), the measure of overall binge drinking captures a type a binge drinking (combination binge drinking or occasions based on the combination of multiple beverage types) that is not necessarily captured by the beverage‐specific measures, if an occasion of binge drinking includes > 1 beverage type. Consequently, the beverage‐specific measures used here are not a precise decomposition of the measure of overall binge drinking. To provide some indication of the proportion of overall binge drinking prevalence that is not captured by the beverage‐specific measures, in supplementary analyses we examined the distribution of the reported set of beverage‐specific types of binge drinking among those who reported overall binge drinking separately by age. We did so to ascertain the proportion of overall binge drinkers who do not report any occasions of beer, wine, or liquor binge drinking.

#### 2.2.2 Cohort Group

Respondents were assigned to one of seven cohort groups based on the year they reached age 30: 1988–1992; 1993–1997; 1998–2002; 2003–2007; 2008–2012; 2013–2017; 2018–2020.

#### 2.2.3 Sex

Sex was self‐reported and measured (i.e., “What is your sex?”) dichotomously at age 18 (Male = 0, Female = 1).

### 2.3 Analytic Plan

Analyses were conducted with Mplus Version 8 (Muthén and Muthén 1998–2017). We used Kline's (2015) guidelines to assess model fit: Root Mean Square Error of Approximation (RMSEA) smaller than 0.05 represents close fit and Comparative Fit Index (CPI) larger than 0.95 indicates excellent fit. Preliminary analyses indicated that historical trends in binge drinking did not vary based on whether individuals were randomly followed up one year versus two years after baseline. Thus, to eliminate unnecessary complexity, the two random halves of each cohort are combined in all analyses. Hereafter, for simplicity we only refer to the even‐numbered ages when referring to the ages that correspond to waves. For analyses of binge drinking prevalence, we used an identity link in conjunction with a robust maximum likelihood sandwich estimator that is robust to non‐normality. We did so because when comparing effects across age, cohort group, and sex we are interested in absolute differences in binge drinking prevalence—or risk differences—which are obtained via an identity link function, as opposed to relative differences in binge drinking prevalence—or odds ratios and risk ratios—which are obtained via a non‐linear logit link function (Cheung 2007; Schechtman 2002; Sinclair and Bracken 1994). Simulation studies have shown (Cheung 2007; Pedroza and Thanh Truong 2016; Ukoumunne et al. 2008) that an identity link function used in conjunction with a robust maximum likelihood sandwich estimator reliably produces unbiased estimates and standard errors of risk difference in binary outcomes.

#### 2.3.1 Aim 1. Delineating Age Trends (Age Main Effects) by Beverage Type

We used a piecewise latent‐growth model (Kline 2015; see black portion of Figure 1) to assess age 18–30 trajectories (i.e., age main effects) of binge drinking. We estimated the following factors: (1) age 18 intercept, (2) age 18–22 linear growth, (3) age 22–26 linear growth, and (4) age 26–30 linear growth. Separate growth models were specified for overall, beer, wine, and liquor binge drinking. We conducted multiple group analyses with sex as a grouping variable to model all parameters separately for women and men. Example Mplus syntax is provided in Supporting Information.

**FIGURE 1:**
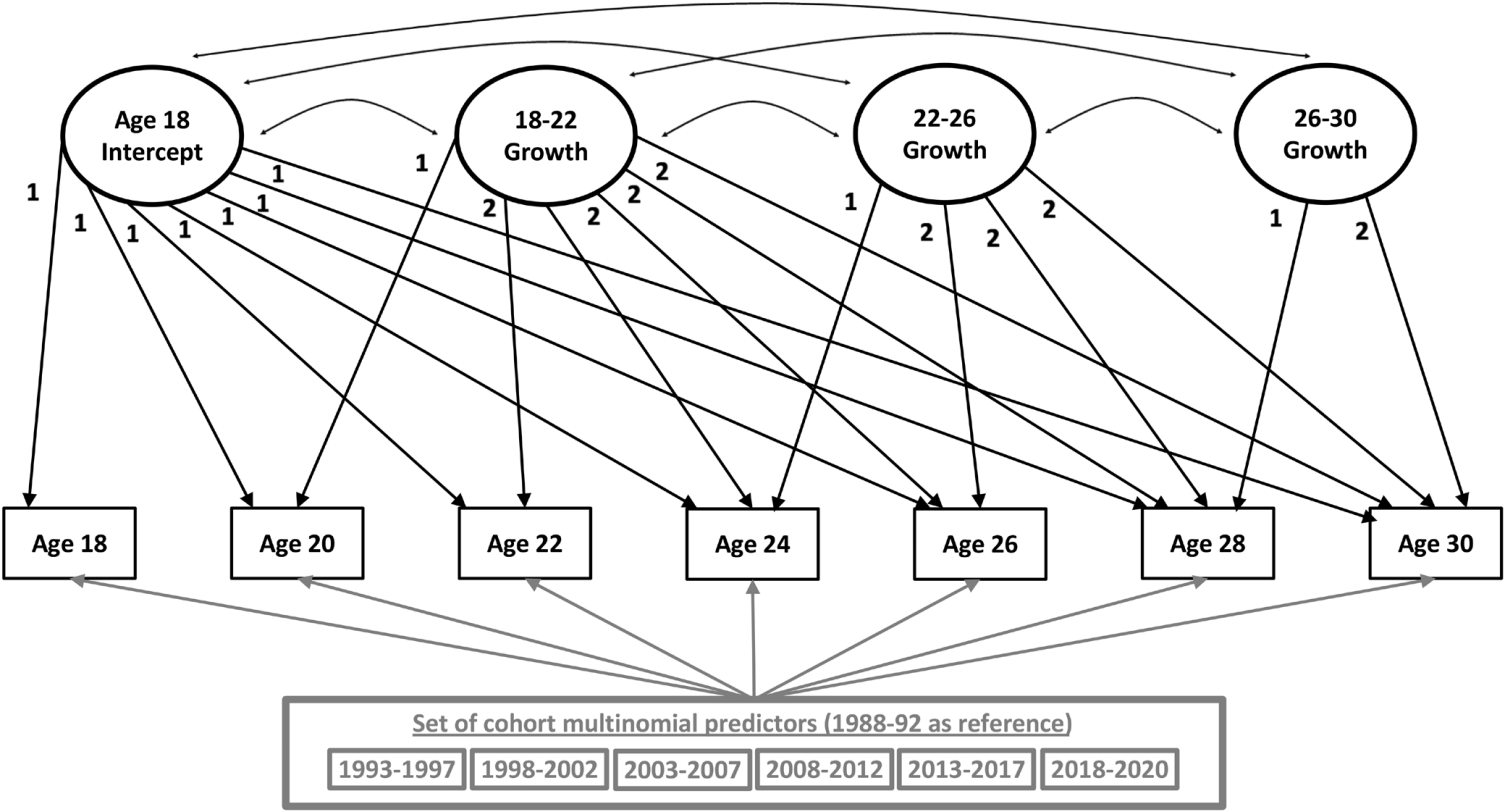
Piecewise latent‐growth model of binge drinking with cohort group as age‐varying predictor.

#### 2.3.2 Aims 2 and 3. Delineating Historical Trends (Cohort Main Effect) and Age‐by‐Historical Interactions (Cohort by Age Interaction) by Beverage Type

We capture age‐varying effects of cohort group by specifying the groups (with the 1988–1992 group as the reference group) as predictors of each binge drinking indicator (See gray portion within Figure 1). At each age, these effects capture whether cohort group is associated with deviations from predicted values of binge drinking based on the overall growth trajectory. Cohort main effects (i.e., Aim 2) are captured by the across‐age average of the effects of cohort group, whereas age‐by‐cohort group interactions (i.e., Aim 3) are captured by across‐age variation in the effects of cohort group. Cohort group main effects (i.e., across‐age averages) were determined by models constraining cohort group effects to be the same across age. We further probed cohort group main effects by using the model constraint command in Mplus (Muthén and Muthén, 1998) to create a new model parameter that captures net‐linear trends across cohort group based on the average of the six lagged differences across the seven cohort groups (i.e., equivalent to fitting a linear trend line through the set of cohort group estimates). The estimates for these new model parameters as well as their standard errors were calculated within Mplus. Age‐by‐cohort group interactions (i.e., across‐age variation in cohort group effects) were determined via chi‐square difference tests (e.g., model fit where effects are constrained to be the same across age is compared to model fit where effects are free to vary across age). For age‐by‐cohort interactions, we again further probed cohort group effects by using the same approach via the model constraint command described earlier for cohort main effects; However, for cohort by age interactions we created new model parameters of net‐linear trends across cohort group separately for each age. Given our use of a robust maximum likelihood estimator, all chi‐square difference tests were tested via the appropriate adjustment equations (Satorra and Bentler 2001). Once again, we conducted multiple group analyses with sex as a grouping variable to model all parameters separately for women and men. Example Mplus syntax is provided in Supporting Information.

## 3 Results

### 3.1 Aim 1: Delineating Age Trends by Beverage Type

Figure 2a,b provide for women and men, respectively, a descriptive summary of age 18–30 trajectories of binge prevalence for each beverage type. Table 1 presents intercept and growth factor estimates for overall, beer, wine, and liquor binge drinking prevalence. The fit of each growth model tested was excellent; thus, given space constraints, fit indices for each model are not discussed but are provided within Table S1.

**FIGURE 2:**
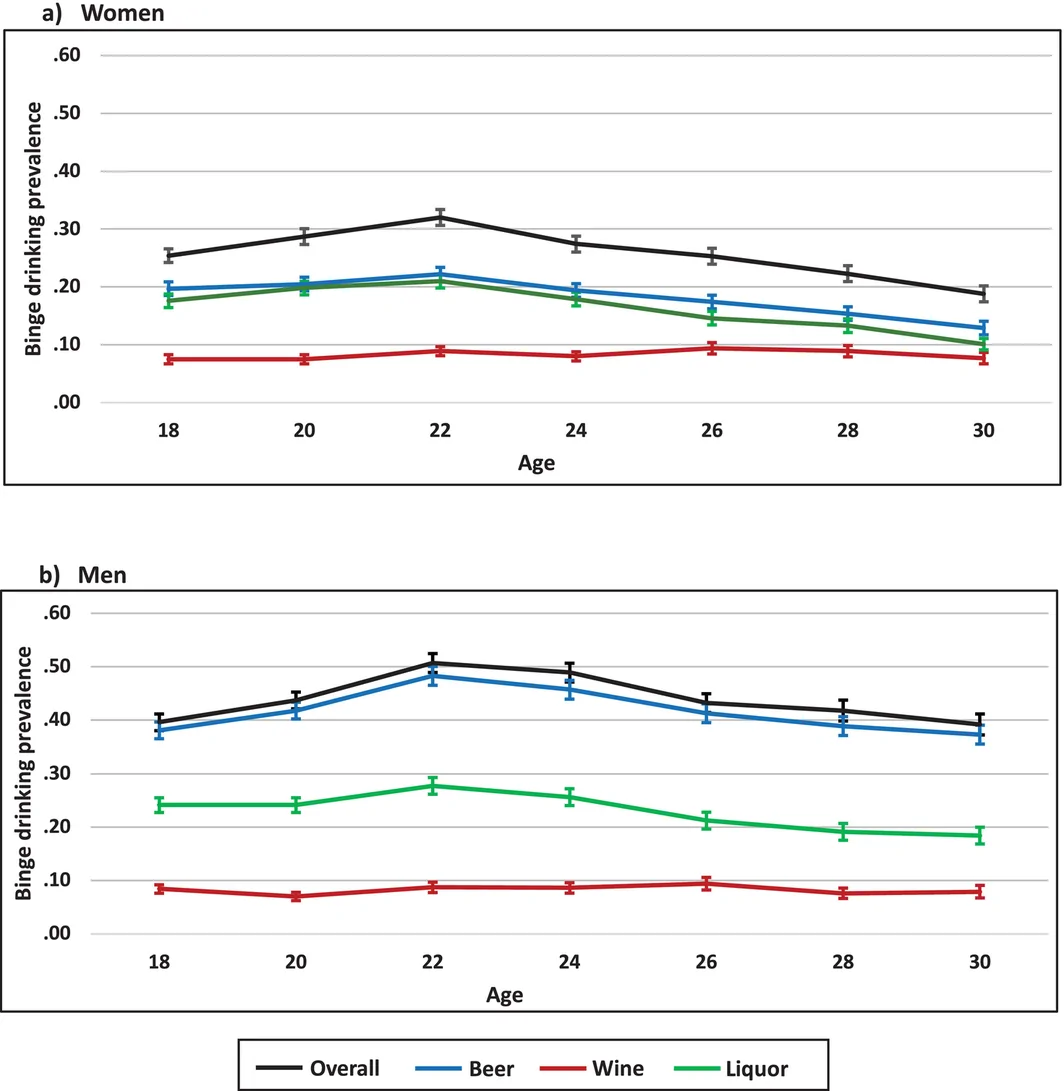
Binge drinking trajectories by beverage type, with 95% error bars. Trajectories are based on weighted means (using combination sample/attrition weights).

**TABLE 1:** Age 18–30 growth in binge drinking prevalence.

Growth factors	Growth factor estimates	
	Women	Men
	Est. (95% CI)	Est. (95% CI)
Overall binge drinking		
Age 18 intercept	0.252 (0.240, 0.264)^a^	0.387 (0.373, 0.401)^a^
Age 18–22 growth	0.032 (0.024, 0.040)^a^	0.060 (0.050, 0.070)^a^
Age 22–26 growth	−0.033 (−0.041, −0.025)^a^	−0.036 (−0.046, −0.026)^a^
Age 26–30 growth	−0.031 (−0.039, −0.023)^a^	−0.025 (−0.035, −0.015)^a^
Beer binge drinking		
Age 18 intercept	0.198 (0.186, 0.210)^a^	0.377 (0.361, 0.393)^a^
Age 18–22 growth	0.011 (0.003, 0.019)^a^	0.051 (0.041, 0.061)^a^
Age 22–26 growth	−0.024 (−0.032, −0.016)^a^	−0.032 (−0.042, −0.022)^a^
Age 26–30 growth	−0.021 (−0.027, −0.015)^a^	−0.022 (−0.032, −0.012)^a^
Wine binge drinking		
Age 18 intercept	0.073 (0.067, 0.079)^a^	0.078 (0.070, 0.086)^a^
Age 18–22 growth	0.005 (−0.001, 0.011)	0.002 (−0.004, 0.008)
Age 22–26 growth	0.004 (−0.002, 0.010)	0.004 (−0.002, 0.010)
Age 26–30 growth	−0.006 (−0.012, 0.000)	−0.007 (−0.013, 0.000)
Liquor binge drinking		
Age 18 intercept	0.177 (0.167, 0.187)^a^	0.234 (0.220, 0.248)^a^
Age 18–22 growth	0.016 (0.008, 0.024)^a^	0.020 (0.010, 0.030)^a^
Age 22–26 growth	−0.031 (−0.039, −0.023)^a^	−0.030 (−0.040, −0.020)^a^
Age 26–30 growth	−0.022 (−0.030, −0.014)^a^	−0.016 (−0.026, −0.006)^a^

*Note:* 95% confidence intervals are provided in parentheses.

^a^ Statistically significant finding (95% confidence interval excludes 0).

Although overall binge drinking prevalence was lower for women than men regardless of age (see Figure 2a,b), for both sexes overall binge drinking prevalence followed an inverted U‐shaped growth pattern (see Table 1) characterized by increases across ages 18–22 (women = 0.032; men = 0.060) and decreases across ages 22–26 (women = −0.033; men = −036) and ages 26–30 (women = −0.031; men = −0.025).

For both women and men, age trends in binge drinking prevalence for beer and liquor generally approximated age trends for overall binge drinking prevalence, as indicated by the congruent pattern of growth estimates for beer, wine, and overall binge drinking in Table 1 and illustrated descriptively in Figure 2a,b, where the trajectories for beer, liquor, and overall binge drinking were largely parallel to one another. In contrast, binge drinking prevalence for wine was largely flat across age for both women and men, as indicated by the non‐significant growth estimates in Table 2 and illustrated descriptively in Figure 2a,b by the largely flat trajectories for wine. Sex differences in overall binge drinking prevalence were primarily a function of beer. Among the three beverage types, the sex difference for beer was consistently the largest by a wide margin regardless of age (see Figure 2).

**TABLE 2:** Cohort main effects (across‐age average) in binge drinking.

Cohort groups	Cohort group estimates	
	Women	Men
	Est. (95% CI)	Est. (95% CI)
Overall binge drinking		
1988–1992 (Ref)	[0.251 (0.231, 0.271)^a^]	[0.424 (0.398, 0.450)^a^]
1993–1997	−0.007 (−0.033, 0.019)	0.000 (−0.036, 0.036)
1998–2002	−0.027 (−0.053, −0.001)^a^	−0.044 (−0.080, −0.008)^a^
2003–2007	−0.014 (−0.042, 0.014)	−0.053 (−0.091, −0.015)^a^
2008–2012	0.037 (0.005, 0.069)^a^	−0.033 (−0.075, 0.009)
2013–2017	0.012 (−0.018, 0.042)	−0.043 (−0.087, 0.001)
2018–2020	0.016 (−0.020, 0.052)	−0.126 (−0.174, −0.078)^a^
Net‐linear trend	0.003 (−0.003, 0.009)	−0.021 (−0.029, −0.013)^a^
Age variation *p*	< 0.001	< 0.001
Beer binge drinking		
1988–1992 (Ref)	[0.193 (0.175, 0.211)^a^]	[0.407 (0.381, 0.433)^a^]
1993–1997	0.023 (−0.001, 0.047)	0.015 (−0.021, 0.051)
1998–2002	−0.007 (−0.031, 0.017)	−0.035 (−0.071, 0.001)
2003–2007	0.000 (−0.026, 0.026)	−0.044 (−0.082, −0.006)^a^
2008–2012	0.024 (−0.004, 0.052)	−0.035 (−0.077, 0.007)
2013–2017	−0.005 (−0.031, 0.021)	−0.038 (−0.080, 0.004)
2018–2020	−0.010 (−0.040, 0.020)	−0.118 (−0.164, −0.072)^a^
Net‐linear trend	−0.002 (−0.008, 0.004)	−0.020 (−0.028, −0.012)^a^
Age variation *p*	< 0.001	< 0.001
Wine binge drinking		
1988–1992 (Ref)	[0.092 (0.080, 0.104)^a^]	[0.101 (0.087, 0.115)^a^]
1993–1997	−0.012 (−0.026, 0.002)	−0.009 (−0.025, 0.007)
1998–2002	−0.040 (−0.054, −0.026)^a^	−0.040 (−0.056, −0.024)^a^
2003–2007	−0.042 (−0.056, −0.028)^a^	−0.040 (−0.056, −0.024)^a^
2008–2012	−0.019 (−0.035, −0.003)^a^	−0.019 (−0.037, −0.001)^a^
2013–2017	−0.015 (−0.031, 0.001)	−0.027 (−0.045, −0.009)^a^
2018–2020	0.004 (−0.018, 0.026)	−0.033 (−0.053, −0.013)^a^
Net‐linear trend	0.001 (−0.003, 0.005)	−0.006 (−0.010, −0.002)^a^
Age variation *p*	< 0.001	< 0.001
Liquor binge drinking		
1988–1992 (Ref)	[0.160 (0.144, 0.176)^a^]	[0.228 (0.208, 0.248)^a^]
1993–1997	−0.012 (−0.030, 0.006)	−0.016 (−0.040, 0.008)
1998–2002	−0.024 (−0.042, −0.006)^a^	−0.038 (−0.064, −0.012)^a^
2003–2007	−0.004 (−0.024, 0.016)	−0.012 (−0.040, 0.016)
2008–2012	0.058 (0.034, 0.082)^a^	0.029 (−0.001, 0.059)
2013–2017	0.064 (0.040, 0.088)^a^	0.071 (0.039, 0.103)^a^
2018–2020	0.074 (0.042, 0.106)^a^	0.033 (−0.009, 0.075)
Net‐linear trend	0.012 (0.006, 0.018)^a^	0.005 (−0.003, 0.013)
Age variation *p*	< 0.001	< 0.001

*Note:* 95% confidence intervals are provided in parentheses. Ref = reference group. For the reference groups, the estimates provided in brackets are intercepts (i.e., mean prevalence values for the reference group).

^a^ Statistically significant finding (95% confidence interval excludes 0). Age variation *p* values are based on chi‐square difference tests (see Table S1 for chi‐square difference tests).

### 3.2 Aim 2. Delineating Historical Trends by Beverage Type

*Cohort main effects* for women and men are presented in Table 2. These cohort main effects capture differences across cohort groups when averaging effects across ages 18–30 (i.e., assume differences across cohort groups are invariant across age). For women, overall binge drinking and beer binge drinking prevalence was relatively stable across cohort groups (net‐linear trends did not differ from zero), whereas wine binge drinking prevalence was curvilinear across cohort groups (although the net‐linear trend did not overlap with zero, relative to the 1988–1992 cohort group, the intermediate cohort groups were lower, whereas more recent cohort groups were equivalent), and liquor binge drinking prevalence increased across cohort groups (the net‐linear trend was positive). For men, prevalence for liquor binge drinking was generally stable across cohort groups (the net‐linear trend did not differ from zero), whereas prevalence for overall binge drinking, beer binge drinking, and wine binge drinking decreased across cohort groups (where the net‐linear trends were all negative), and for both overall and beer binge drinking, the decrease relative to the 1988–1992 reference cohort group was particularly evident for the 2018–2020 cohort group.

### 3.3 Aim 3. Delineating Age‐Varying Historical Trends by Beverage Type

*Age‐by‐cohort group interactions* for women and men are indicated in Table 2 by age variation *p* values, where statistically significant *p* values indicate that a model allowing differences across cohort group to vary by age fit significantly better than a model where cohort group differences were constrained to be the same across age (see Table S1 for model comparisons). As indicated in Table 2, for both women and men, age‐by‐cohort group interactions were evident for overall, beer, wine, and liquor binge drinking. Age‐specific effects for cohort group differences and for net‐linear trends across cohort groups are presented for women and men, respectively, in Figures 3 and 4 and in Tables S2 and S3.

**FIGURE 3:**
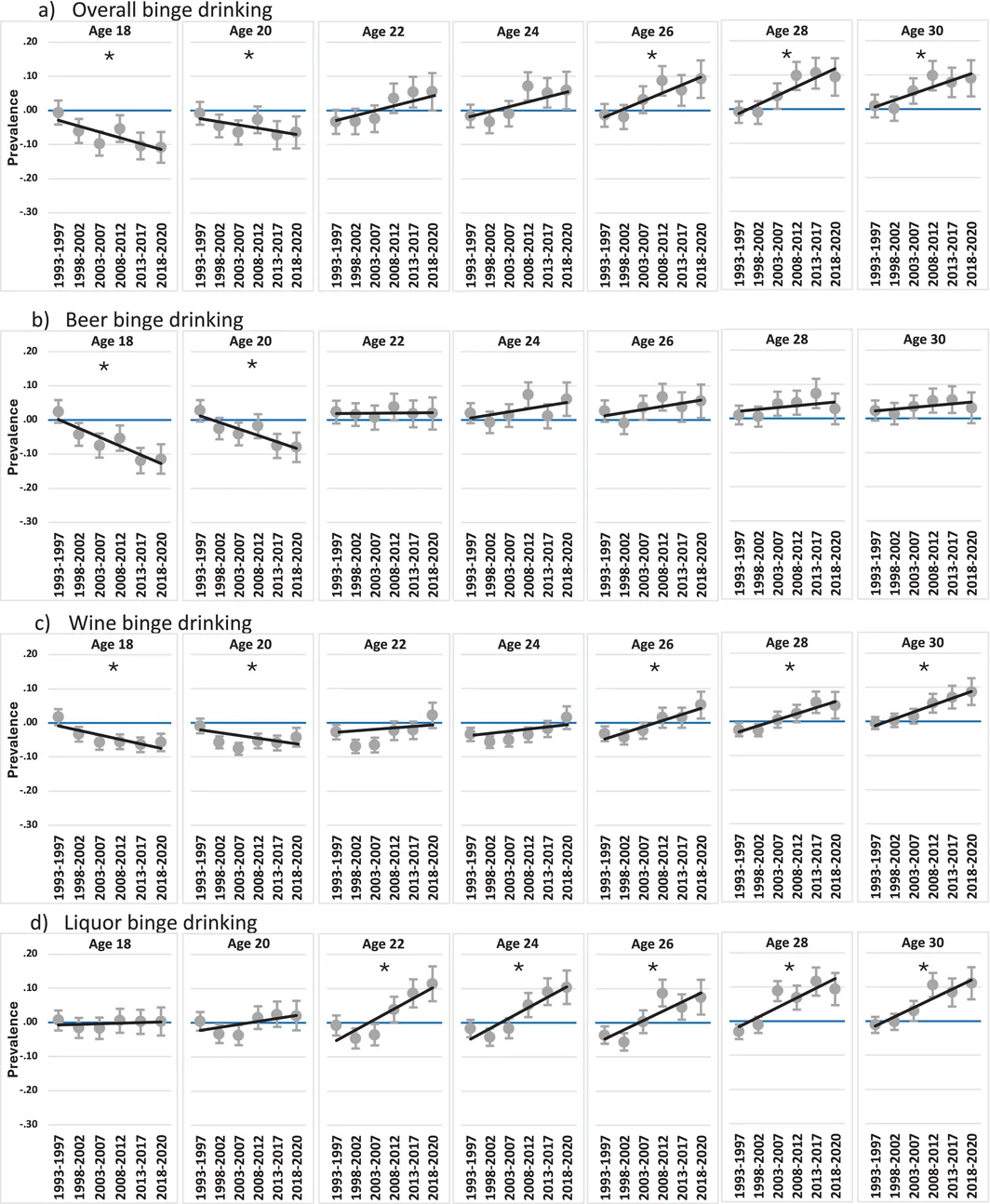
Among women, age‐varying effects of cohort group on binge drinking prevalence by beverage type, with the 1988–1992 historical group as reference. Error bands correspond to 95% confidence intervals. Solid black lines depict estimates of net‐linear trends across cohort groups. *Indicates net‐linear estimate is significant at 0.05 level.

**FIGURE 4:**
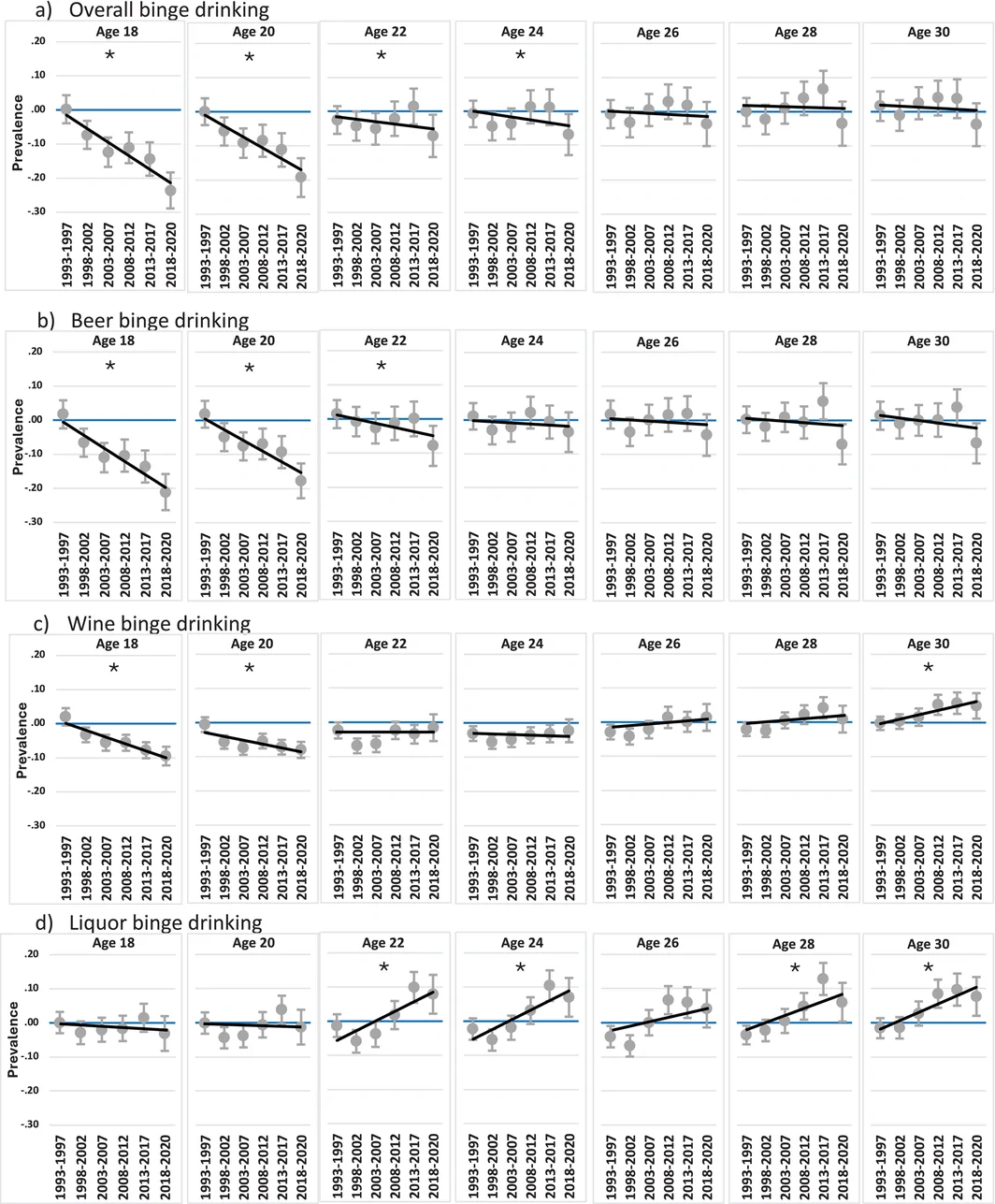
Among men, age‐varying effects of historical group on binge drinking prevalence by beverage type, with the 1988–1992 historical group as reference. Error bands correspond to 95% confidence intervals. Solid black lines depict estimates of net‐linear trends across cohort groups. *Indicates net‐linear estimate is significant at 0.05 level.

For women, cohort group differences in overall binge drinking prevalence varied by age in a manner that was consistent with a historical‐developmental reversal (Figure 3a; Table S2). Specifically, across the most distant (1988–1992) and recent (2018–2020) cohort group examined here (hereafter referred to as “across cohort group”), overall binge drinking prevalence for women decreased at ages 18 and 20 (where net‐linear trends were negative), remained flat at ages 22 and 24 (where net‐linear trends did not differ from zero), and increased at ages 26, 28, and 30 (where net‐linear trends were positive).

Beverage types not only differed for women in the degree of their contribution to the historical‐developmental reversal but also as to where within the age 18–30 age band (i.e., decreases across cohort group at the younger portion of the age band versus increases across cohort group at the older portion of the age band) they contributed to the reversal. Decreases across cohort group at ages 18 and 20 were a function of beer (where net‐linear trends were negative; Figure 4b, Table S2) and wine (where net‐linear trends were negative; Figure 4c, Table S2). At ages 18 and 20, binge drinking prevalence for liquor was largely flat across cohort group (where net‐linear trends did not differ from zero; Figure 4d, Table S2). Eventual increases across cohort group in binge drinking prevalence at ages 26, 28, and 30 were a function of liquor and wine. Binge drinking prevalence for liquor increased across cohort group at ages 22–30 (where net‐linear trends were positive; Figure 4d, Table S2). Binge drinking prevalence for wine increased across cohort group at ages 26–30 (where net‐linear trends were positive; Figure 4c, Table S2). For beer, there was limited and mixed evidence for increases across cohort group among the older portion of the 18–30 age band (Figure 3b); at some ages (i.e., ages 24, 26, and 28) there was evidence of higher levels among intermediate cohort groups, but at no age was there evidence of increases across cohort group (for all ages net‐linear trends did not differ from zero). In summary, decreases across cohort group at the younger end of the 18–30 age band were primarily a function of beer and wine, whereas increases across historical group at the older end of the 18–30 age band were primarily a function of liquor and wine.

For men, historical trends in overall binge drinking prevalence varied by age but in a manner indicating that declines across cohort group at the younger portion of the 18–30 age band were lost (as opposed to being reversed) at the older portion of the 18–30 age band (Figure 4a; Table S3). Specifically, across cohort group, overall binge drinking prevalence for men decreased at ages 18 through 24 (where net‐linear trends were negative, particularly so at ages 18 and 20) but remained flat at ages 26 through 30 (where net‐linear trends did not differ from zero).

Similar to women, decreases across cohort group at the younger portion of the age 18–30 age band were evident for beer and wine; however, for men these decreases were stronger for beer compared to wine (as evidenced by non‐overlapping 95% confidence intervals for net‐linear trends in beer and wine at ages 18 and 20, Table S3). Binge drinking prevalence for beer decreased across cohort group at ages 18, 20, and 22 (where net‐linear trends were negative; Figure 4b, Table S3). Binge drinking prevalence for wine decreased across cohort group at ages 18–20 (where net‐linear trends were negative; Figure 4c, Table S3). Also similar to women, increases across cohort group at the older portion of the age 18–30 age band were evident for liquor and wine. Binge drinking prevalence for liquor increased across cohort group at ages 22, 24, 28, and 30 (where net‐linear trends were positive; Figure 4d, Table S3). Binge drinking prevalence for wine increased across cohort group at age 30 (where net‐linear trends were positive; Figure 4c, Table S3). Thus, although overall binge drinking prevalence did not increase across cohort group at the older portion of the 18–30 age band, binge drinking prevalence for both liquor and wine did.

### 3.4 Supplementary Analyses

The distributions of the types of beverage‐specific binge drinking reported among those who reported overall binge drinking over the last 14 days are shown separately by age in Table S4. The percentage of binge drinkers who do not report any occasions of beer, wine, or liquor binge drinking did not vary by age and was relatively small (ranging from 7.4% to 10%). Generally, among binge drinkers around 45%–55% of respondents reported binge drinking exclusive to one beverage type, although this percentage increased with age due to increases across age in beer‐only and wine‐only binge drinking as well as decreases across age in beer/liquor and beer/liquor/wine binge drinking. Due to the nature of the measures used here, the combination of multiple beverage types of binge drinking over the last 14 days does not indicate combination binge drinking or the combination of multiple beverage types within a single occasion of binge drinking (e.g., the engagement in beer and wine binge drinking over the last 14 days could represent two separate occasions of binge drinking that are each homogeneous to the beverage type consumed, or could represent one combination occasion with binging of both beer and wine).

## 4 Discussion

Available research indicates that young adult binge drinking follows an inverted U‐shaped pattern across age, and, although there is evidence of decreasing levels among more recent cohorts of young adults (Patrick et al. 2025), over the last several decades young adult binge drinking has remained relatively stable historically (Grucza et al. 2018; Jager et al. 2023; Kerr et al. 2009) among young adults as an aggregate group. Nevertheless, young adult binge drinking has varied markedly within young adulthood with prevalence *decreasing* historically at the onset of young adulthood (i.e., age 18) but *increasing* historically by the end of young adulthood (i.e., age 30) (Jager et al. 2015, 2023; Patrick et al. 2019). The current study, which seeks to complement this existing work focused on overall binge drinking by disaggregating age and historical trends in binge drinking by beer, wine, and liquor, offers three main conclusions. First, young adult age trends in binge drinking were not uniform across beverage type, with beer and liquor trends largely mirroring inverted U‐shaped trends for overall binge drinking but wine trends proving comparably flat across age. Second, young adult historical trends in binge drinking were not uninform across beverage type or sex: for women, liquor increased across cohort group, whereas beer and wine trends mirrored those for overall binge drinking, and remained stable; however, for men, liquor trends remained stable, whereas beer and wine trends decreased historically and mirrored those for overall binge drinking. Third, young adult historical trends in binge drinking varied sharply by age and differentially so by beverage type. Specifically, for both sexes, historical decreases in overall binge drinking at the outset of young adulthood were primarily a product of historical decreases in beer and wine. However, due to historical increases in liquor and wine during the latter portion of young adulthood, for both sexes these initial decreases in overall binge drinking were not maintained through the end of young adulthood (i.e., age 30).

Overlaying existing work focused on overall binge drinking, which helps clarify the ages within young adulthood the risks associated with binge drinking are particularly localized and how the location of those risks ebbs and flows across historical time, the present study clarifies how these risks further ebb and flow by beverage type. Findings also suggest that an effective strategy maintaining and extending the early young adulthood declines in binge drinking through age 30 (which were primarily lost by age 30 due to liquor and wine) would be expanded prevention and policy efforts that specifically target liquor and wine consumption.

### 4.1 Implications for the Timing and Concentration of Young Adult Alcohol‐Related Harms

Given the across‐age reversal in historical effects for women found here and found previously (Jager et al. 2015, 2023), more recent historical groups of women may face fewer harms associated with overall binge drinking at the outset of young adulthood yet face more harms associated with overall binge drinking during the latter half young adulthood. Going beyond this prior work, here we further disaggregated young adult historical trends by beverage type, and consequently we were also able to identify which beverage types were the primary drivers of these historical trends. Consequently, our findings not only indicate that timing of alcohol‐related harms associated with overall drinking have been potentially displaced to older ages within the 18–30 age band for women (i.e., on account of the across‐age reversal in overall binge drinking), but also that those increased risks at older ages are particularly concentrated among liquor and wine (i.e., on account of historical increases in liquor and wine but not beer during the latter portion of young adulthood for women).

Unlike past work (Jager et al. 2015, 2023) indicating an across‐age reversal in historical trends for young adult men, here we merely found that historical decreases at age 18 for men were lost by age 30, where overall binge drinking prevalence was historically stable. A possible explanation for the inconsistency in findings is that this past work did not include the most recent cohorts examined here (e.g., 2018–2020 cohort group). Like past work, our findings were consistent with modest historical increases at ages 28 and 30 for men up until the 2018–2020 cohort group, which evidenced trends (albeit non‐significant) in the opposite direction. Nevertheless, our findings indicating that historical declines at age 18 for men were lost by age 30 indicate that more recent historical groups of men may face reduced alcohol‐related harms relative to past historical groups, but only during the younger portion of the 18–30 age band, where historical declines in beer and wine binge drinking but not liquor were evident. Although at the older portion of the 18–30 age band overall binge drinking prevalence was historical stable for men, both liquor and wine binge drinking increased historically. Consequently, among more recent historical groups of men the alcohol‐related harms faced at the older portion of the 18–30 age band may be more concentrated among liquor and wine relative to past historical groups.

Thus, for both women and men, liquor and wine binge drinking evidence historical increases specifically during the older portion of the age 18–30 age band. These trends not only have Implications for the public health risks associated with binge drinking, they also point to the need for intervention and policy efforts (Naimi et al. 2007) that target these specific beverages. With respect to public health risks, increases in liquor and wine but not beer binge drinking necessarily translates into liquor and wine comprising a greater overall share of binge drinking. This may elevate the overall risks associated with binge drinking, given that overall liquid volume tends to be lower for liquor and wine beverages compared to beer, whereas both alcohol volume and across‐drink variability in alcohol volume tends to be higher for liquor and wine beverages compared to beer (Gual et al. 1999; Kerr et al. 2005, 2008; Lemmens 1994). With respect to intervention and policy, there is ample opportunity to expand and refine efforts focused on wine and liquor, given that policies related to price and availability across US state often differ by beverage type. For example, alcohol taxation policies have historically differentiated between beer, wine, and liquor. Although there is variation across state, wine and especially liquor are generally taxed at a higher level than beer per ounce of alcohol (Naimi et al. 2018), although differences are modest when considering excise taxes which are the predominant form of alcohol taxation in the United States. Additional tax increases on liquor and wine would predict lower rates of consumption given that evidence consistently shows a strong relationship between taxation and consumption (Subbaraman et al. 2020), and liquor and wine are more price elastic than beer (Guindon et al. 2022), emphasizing that price increases are likely to generate population level reductions. A range of additional alcohol policies are beverage specific and, thus, can be further refined to target liquor and wine, including point‐of‐sale restrictions. Although there are significant point‐of‐sale restrictions on liquor in most states (e.g., no sales in grocery stores, gas stations, convenience stores), such restrictions are not ubiquitous in all states and wine can be sold in grocery stores in most states, thus additional restrictions should be considered to reduce rising consumption (Hahn et al. 2010).

### 4.2 Beverage‐Specific Contributions to the Historical Narrowing of Sex Differences in Binge Drinking

Consistent with prior work (Keyes et al. 2019; Patrick et al. 2025), our findings indicate that the size of the sex difference in young adult binge drinking narrowed across historical time. Specifically, because we found that overall binge drinking among all young adults (i.e., cohort main effects among ages 18–30 combined) decreased across cohort group for men but remained stable across cohort group for women, it necessarily follows that the size of the sex difference (i.e., the degree to which drinking among young adult men surpasses drinking among young adult women) narrowed across cohort group. Extending beyond prior work, which did not disaggregate young adult historical trends by beverage type, our findings more specifically indicate that the narrowing of sex differences over historical time was primarily a function of two patterns: (1) historical declines in beer and wine binge drinking for only young adult men, and (2) historical increases in liquor binge drinking for only young adult women. The pattern found for beer and wine binge drinking—historical decreases for men only—is likely advantageous for public health. However, the pattern found for liquor binge drinking—historical increases only for women—may be more troublesome in terms of public health and warrants greater attention in terms of both research and public policy.

### 4.3 Strengths and Limitations

Important strengths of this study include the use of US national multi‐cohort, multi‐wave panel data, which permitted the delineation of binge drinking as a moving target developmentally and historically, and the analytical approach, which was sensitive to how historical effects fluctuate (i.e., interact) with age. This study also has important limitations. First, and as noted above, the beverage‐specific measures used here do not capture combination binge drinking or binge drinking characterized by combinations of beverage types (e.g., three beers and two cocktails) and consequently are not a precise decomposition of the measure of overall binge drinking. Nevertheless, supplementary analyses indicated prevalence of overall binge drinking was generally well captured (only around 8% of individuals who endorsed at least one occasion of binge drinking indicated no beverage‐specific beer, wine, or liquor binge drinking), indicating that there were relatively few binge drinkers who exclusively engage in combination binge drinking. However, the prevalence of combination binge drinking is likely higher than the rate of exclusive combine binge drinking, because there are individuals who engage in combination binge drinking but not exclusively (i.e., they also engage in beverage‐specific binge drinking). For example, prior work focused on disaggregating respondents' last occasion of binge drinking among all US adults (Naimi et al. 2007) found that 41% were characterized as combination binge drinking, second only to beer‐only binge drinking (45%). Consequently, to the extent data allow, future work incorporating combination binge drinking is needed in order to fully decompose age and historical trends of overall binge drinking by beverage type. Second, this study relies on self‐report data, introducing the possibility of reporting bias. Third, the sampling frame excluded high‐school dropouts. Fourth, for binge drinking we used a general measure of 5+ drinks for both men and women rather than sex‐specific levels of 4+ for women and 5+ for men (e.g., CDC 2012). The use of the same 5+ threshold for men and women may understate sex differences in binge drinking, to the extent that 4+ drinks for women is capturing the same level of harm as 5+ drinks for men.

## 5 Conclusions and Future Directions

This study provides the first detailed examination of beverage‐specific binge drinking trajectories across young adulthood and over historical time in the United States. Findings reveal that although beer and liquor binge drinking follow inverted U‐shaped age patterns, wine binge drinking remains relatively flat across age. Historically over the past 30 years, overall binge drinking has declined for men but remained stable for women, masking important beverage‐specific shifts. For women, historical declines at age 18 driven by declining beer and wine binge drinking contrast with increases by age 30, largely driven by rising liquor and wine binge drinking. For men, historical declines at age 18 were driven primarily by declining beer binge drinking and secondarily declining wine binge drinking and offset by age 30 due to rising liquor and wine binge drinking. These results underscore the importance of distinguishing beverage types when tracking alcohol use trends. Future research should explore the social, policy, and marketing factors underlying liquor and wine increases and assess how these shifts may alter the timing and burden of alcohol‐related harms across the life course.

## Funding

This work was supported by R01‐AA026861 from the National Institute on Alcohol Abuse and Alcoholism, and R01‐DA001411 and R01‐DA016575 from the National Institute of Drug Abuse. The National Institute on Alcohol Abuse and Alcoholism and the National Institute of Drug Abuse had no further role in the study design; in the collection, management, analysis, and interpretation of the data; in the writing of the manuscript; or in the decision to submit the paper for publication. The views expressed in this article are those of the authors and do not necessarily reflect the views of the funders.

## Conflicts of Interest

The authors declare no conflicts of interest.

## Supporting information


**Data S1:** Supporting Information.

**Data S2:** Supporting Information.

**Table S1:** Model fit indices and corresponding Santora‐Bentler corrected chi‐square difference tests.
**Table S2:** Age‐varying effects of cohort group on binge drinking prevalence among women, by beverage type.
**Table S3:** Age‐varying effects of cohort group on binge drinking prevalence among men, by beverage type.
**Table S4:** The distribution of reported beverage‐specific types of binge drinking among those who reported overall binge drinking by age.

## Data Availability

The data that support the findings of this study are openly availablein Monitoring the Future at https://monitoringthefuture.org/results/data‐access/.
