# Supplementary material for: Age 18–30 Trajectories of Beverage‐Specific Binge Drinking Prevalence Over 30 Years for Men and Women in the United States
Source: Alcohol Clin Exp Res (Hoboken). 2026 Oct 15;50(10):e70420. doi: 10.1111/acer.70420 (PMC13673115; doi:10.1111/acer.70420)
Supplement: Supplementary file 1 [file ACER-50-0-s001.pdf]

```
TITLE:
   SUPPLEMENTAL MATERIALS
   EXAMPLE SYNTAX FOR AIM 2 (Cohort Main effects) and Aim 3 (Age by Cohort Interactions)

   Piece-wise latent growth model of binge drinking prevalence
   with cohort groups as age-varying predictors of binge drinking
   indicators

   Biological sex included as grouping variable

   Model constraint command used to estimate gender differences


DATA:
    FILE IS [FILE NAME HERE]'
    FORMAT IS [FORMAT STATEMENT HERE];

  VARIABLE:
    NAMES ARE

     MTFID
     cPAWab !combination sample/attrition weight
     binge18 binge20 binge22 binge24 binge26 binge28 binge30 !Binge drinking indicators

     sex; !Biological sex measure
     coh7680 !Reference cohort group
     coh8185 coh8690 coh9195 coh9600 coh0105 coh0608 !non-reference cohort groups



USEVAR binge18 binge20 binge22 binge24 binge26 binge28 binge30
       coh8185 coh8690 coh9195 coh9600 coh0105 coh0608; !cohort7680 as reference

GROUPING is sex (0 = male 1 = female);
```

```
MISSING ARE .;

weight cPAWab;


ANALYSIS:
type = Meanstructure;
estimator = MLR;
Iterations = 100000;


MODEL:

 !!! Specification of growth factors!!!
 Ibinge by binge18@1 binge20@1 binge22@1 binge24@1 binge26@1 binge28@1 binge30@1;
 L1binge by binge18@0 binge20@1 binge22@2 binge24@2 binge26@2 binge28@2 binge30@2;
 L2binge by binge18@0 binge20@0 binge22@0 binge24@1 binge26@2 binge28@2 binge30@2;
 L3binge by binge18@0 binge20@0 binge22@0 binge24@0 binge26@0 binge28@1 binge30@2;

 [Ibinge];
 [L1binge];
 [L2binge];
 [L3binge];

 Ibinge;
 L1binge;
 L2binge;
 L3binge;


 !specifying Covariances among growth factors
 Ibinge with L1binge L2binge L3binge;
 L1binge with L2binge L3binge;
 L2binge with L3binge;

 !Fixing means of growth factor indicators to zero
 [binge18@0]; [binge20@0]; [binge22@0];
 [binge24@0]; [binge26@0]; [binge28@0]; [binge30@0];
```

```
 !Specifying the variances of the growth factor indicators
  binge18; binge20; binge22;
  binge24; binge26; binge28; binge30;


!!!!!!!!!!!!!!!!!!!!!!!!!!!!!!!!!!!!!!!!!!!!!!!!!!!!!!!!!!!!!!!
!Specifying cohort categoricals as predictor of residuals
!!!!!!!!!!!!!!!!!!!!!!!!!!!!!!!!!!!!!!!!!!!!!!!!!!!!!!!!!!!!!!!!
!Estimates meains of cohort categoricals
[coh8185]; [coh8690]; [coh9195];
[coh9600]; [coh0105]; [coh0608];

!Regressing each binge indicator on set of cohort categoricals, with coh7680 as reference
binge18 on coh8185;
binge18 on coh8690;
binge18 on coh9195;
binge18 on coh9600;
binge18 on coh0105;
binge18 on coh0608;

binge20 on coh8185;
binge20 on coh8690;
binge20 on coh9195;
binge20 on coh9600;
binge20 on coh0105;
binge20 on coh0608;

binge22 on coh8185;
binge22 on coh8690;
binge22 on coh9195;
binge22 on coh9600;
binge22 on coh0105;
binge22 on coh0608;

binge24 on coh8185;
binge24 on coh8690;
binge24 on coh9195;
```

```
binge24 on coh9600;
binge24 on coh0105;
binge24 on coh0608;

binge26 on coh8185;
binge26 on coh8690;
binge26 on coh9195;
binge26 on coh9600;
binge26 on coh0105;
binge26 on coh0608;

binge28 on coh8185;
binge28 on coh8690;
binge28 on coh9195;
binge28 on coh9600;
binge28 on coh0105;
binge28 on coh0608;

binge30 on coh8185;
binge30 on coh8690;
binge30 on coh9195;
binge30 on coh9600;
binge30 on coh0105;
binge30 on coh0608;

!Specifying covariances among cohort group categoricals
coh8185 with coh8690 coh9195 coh9600 coh0105 coh0608;
coh8690 with coh9195 coh9600 coh0105 coh0608;
coh9195 with coh9600 coh0105 coh0608;
coh9600 with coh0105 coh0608;
coh0105 with coh0608;

!Fixing covariances between cohort group categoricals and growth factors to zero
coh8185 on Ibinge@0;
coh8185 on L1binge@0;
coh8185 on L2binge@0;
coh8185 on L3binge@0;
```

```
coh8690 on Ibinge@0;
coh8690 on L1binge@0;
coh8690 on L2binge@0;
coh8690 on L3binge@0;

coh9195 on Ibinge@0;
coh9195 on L1binge@0;
coh9195 on L2binge@0;
coh9195 on L3binge@0;

coh9600 on Ibinge@0;
coh9600 on L1binge@0;
coh9600 on L2binge@0;
coh9600 on L3binge@0;

coh0105 on Ibinge@0;
coh0105 on L1binge@0;
coh0105 on L2binge@0;
coh0105 on L3binge@0;

coh0608 on Ibinge@0;
coh0608 on L1binge@0;
coh0608 on L2binge@0;
coh0608 on L3binge@0;

Model Male:

 !!! Specification of growth factors!!!
 Ibinge by binge18@1 binge20@1 binge22@1 binge24@1 binge26@1 binge28@1 binge30@1;
 L1binge by binge18@0 binge20@1 binge22@2 binge24@2 binge26@2 binge28@2 binge30@2;
 L2binge by binge18@0 binge20@0 binge22@0 binge24@1 binge26@2 binge28@2 binge30@2;
 L3binge by binge18@0 binge20@0 binge22@0 binge24@0 binge26@0 binge28@1 binge30@2;

 [Ibinge](m1);
 [L1binge](m2);
 [L2binge](m3);
 [L3binge](m4);
```

```
 Ibinge;
 L1binge;
 L2binge;
 L3binge;


 !specifying Covariances among growth factors
 Ibinge with L1binge L2binge L3binge;
 L1binge with L2binge L3binge;
 L2binge with L3binge;

 !Fixing means of growth factor indicators to zero
 [binge18@0]; [binge20@0]; [binge22@0];
 [binge24@0]; [binge26@0]; [binge28@0]; [binge30@0];

 !Specifying the variances of the growth factor indicators
  binge18; binge20; binge22;
  binge24; binge26; binge28; binge30;


!!!!!!!!!!!!!!!!!!!!!!!!!!!!!!!!!!!!!!!!!!!!!!!!!!!!!!!!!!!!!!!!
!Specifying cohort categoricals as predictor of residuals
!!!!!!!!!!!!!!!!!!!!!!!!!!!!!!!!!!!!!!!!!!!!!!!!!!!!!!!!!!!!!!!!!
!Estimates meains of cohort categoricals
[coh8185]; [coh8690]; [coh9195];
[coh9600]; [coh0105]; [coh0608];

!Regressing each binge indicator on set of cohort categoricals, with coh7680 as reference
binge18 on coh8185(m5);
binge18 on coh8690(m6);
binge18 on coh9195(m7);
binge18 on coh9600(m8);
binge18 on coh0105(m9);
binge18 on coh0608(m10);

binge20 on coh8185(m11);
binge20 on coh8690(m12);
binge20 on coh9195(m13);
```

```
binge20 on coh9600(m14);
binge20 on coh0105(m15);
binge20 on coh0608(m16);

binge22 on coh8185(m17);
binge22 on coh8690(m18);
binge22 on coh9195(m19);
binge22 on coh9600(m20);
binge22 on coh0105(m21);
binge22 on coh0608(m22);

binge24 on coh8185(m23);
binge24 on coh8690(m24);
binge24 on coh9195(m25);
binge24 on coh9600(m26);
binge24 on coh0105(m27);
binge24 on coh0608(m28);

binge26 on coh8185(m29);
binge26 on coh8690(m30);
binge26 on coh9195(m31);
binge26 on coh9600(m32);
binge26 on coh0105(m33);
binge26 on coh0608(m34);

binge28 on coh8185(m35);
binge28 on coh8690(m36);
binge28 on coh9195(m37);
binge28 on coh9600(m38);
binge28 on coh0105(m39);
binge28 on coh0608(m40);

binge30 on coh8185(m41);
binge30 on coh8690(m42);
binge30 on coh9195(m43);
binge30 on coh9600(m44);
binge30 on coh0105(m45);
binge30 on coh0608(m46);
```

```
!Specifying covariances among cohort group categoricals
coh8185 with coh8690 coh9195 coh9600 coh0105 coh0608;
coh8690 with coh9195 coh9600 coh0105 coh0608;
coh9195 with coh9600 coh0105 coh0608;
coh9600 with coh0105 coh0608;
coh0105 with coh0608;

!Fixing covariances between cohort group categoricals and growth factors to zero
coh8185 on Ibinge@0;
coh8185 on L1binge@0;
coh8185 on L2binge@0;
coh8185 on L3binge@0;

coh8690 on Ibinge@0;
coh8690 on L1binge@0;
coh8690 on L2binge@0;
coh8690 on L3binge@0;

coh9195 on Ibinge@0;
coh9195 on L1binge@0;
coh9195 on L2binge@0;
coh9195 on L3binge@0;

coh9600 on Ibinge@0;
coh9600 on L1binge@0;
coh9600 on L2binge@0;
coh9600 on L3binge@0;

coh0105 on Ibinge@0;
coh0105 on L1binge@0;
coh0105 on L2binge@0;
coh0105 on L3binge@0;

coh0608 on Ibinge@0;
coh0608 on L1binge@0;
coh0608 on L2binge@0;
coh0608 on L3binge@0;
```

```
Model Female:

!!! Specification of growth factors!!!
 Ibinge by binge18@1 binge20@1 binge22@1 binge24@1 binge26@1 binge28@1 binge30@1;
 L1binge by binge18@0 binge20@1 binge22@2 binge24@2 binge26@2 binge28@2 binge30@2;
 L2binge by binge18@0 binge20@0 binge22@0 binge24@1 binge26@2 binge28@2 binge30@2;
 L3binge by binge18@0 binge20@0 binge22@0 binge24@0 binge26@0 binge28@1 binge30@2;

 [Ibinge](f1);
 [L1binge](f2);
 [L2binge](f3);
 [L3binge](f4);

 Ibinge;
 L1binge;
 L2binge;
 L3binge;


 !specifying Covariances among growth factors
 Ibinge with L1binge L2binge L3binge;
 L1binge with L2binge L3binge;
 L2binge with L3binge;

 !Fixing means of growth factor indicators to zero
 [binge18@0]; [binge20@0]; [binge22@0];
 [binge24@0]; [binge26@0]; [binge28@0]; [binge30@0];

 !Specifying the variances of the growth factor indicators
  binge18; binge20; binge22;
  binge24; binge26; binge28; binge30;


!!!!!!!!!!!!!!!!!!!!!!!!!!!!!!!!!!!!!!!!!!!!!!!!!!!!!!!!!!!!!!!!
!Specifying cohort categoricals as predictor of residuals
!!!!!!!!!!!!!!!!!!!!!!!!!!!!!!!!!!!!!!!!!!!!!!!!!!!!!!!!!!!!!!!!!
!Estimates meains of cohort categoricals
```

```
[coh8185]; [coh8690]; [coh9195];
[coh9600]; [coh0105]; [coh0608];

!Regressing each binge indicator on set of cohort categoricals, with coh7680 as reference
binge18 on coh8185(f5);
binge18 on coh8690(f6);
binge18 on coh9195(f7);
binge18 on coh9600(f8);
binge18 on coh0105(f9);
binge18 on coh0608(f10);

binge20 on coh8185(f11);
binge20 on coh8690(f12);
binge20 on coh9195(f13);
binge20 on coh9600(f14);
binge20 on coh0105(f15);
binge20 on coh0608(f16);

binge22 on coh8185(f17);
binge22 on coh8690(f18);
binge22 on coh9195(f19);
binge22 on coh9600(f20);
binge22 on coh0105(f21);
binge22 on coh0608(f22);

binge24 on coh8185(f23);
binge24 on coh8690(f24);
binge24 on coh9195(f25);
binge24 on coh9600(f26);
binge24 on coh0105(f27);
binge24 on coh0608(f28);

binge26 on coh8185(f29);
binge26 on coh8690(f30);
binge26 on coh9195(f31);
binge26 on coh9600(f32);
binge26 on coh0105(f33);
binge26 on coh0608(f34);
```

```
binge28 on coh8185(f35);
binge28 on coh8690(f36);
binge28 on coh9195(f37);
binge28 on coh9600(f38);
binge28 on coh0105(f39);
binge28 on coh0608(f40);

binge30 on coh8185(f41);
binge30 on coh8690(f42);
binge30 on coh9195(f43);
binge30 on coh9600(f44);
binge30 on coh0105(f45);
binge30 on coh0608(f46);

!Specifying covariances among cohort group categoricals
coh8185 with coh8690 coh9195 coh9600 coh0105 coh0608;
coh8690 with coh9195 coh9600 coh0105 coh0608;
coh9195 with coh9600 coh0105 coh0608;
coh9600 with coh0105 coh0608;
coh0105 with coh0608;

!Fixing covariances between cohort group categoricals and growth factors to zero
coh8185 on Ibinge@0;
coh8185 on L1binge@0;
coh8185 on L2binge@0;
coh8185 on L3binge@0;

coh8690 on Ibinge@0;
coh8690 on L1binge@0;
coh8690 on L2binge@0;
coh8690 on L3binge@0;

coh9195 on Ibinge@0;
coh9195 on L1binge@0;
coh9195 on L2binge@0;
coh9195 on L3binge@0;
```

```
coh9600 on Ibinge@0;
coh9600 on L1binge@0;
coh9600 on L2binge@0;
coh9600 on L3binge@0;

coh0105 on Ibinge@0;
coh0105 on L1binge@0;
coh0105 on L2binge@0;
coh0105 on L3binge@0;

coh0608 on Ibinge@0;
coh0608 on L1binge@0;
coh0608 on L2binge@0;
coh0608 on L3binge@0;


MODEL CONSTRAINT:
!!!!!!!!creating new model parameters that capture net linear trends across cohort group !!!!!

NEW (
!Names of net linear trends for men - see below for specifications
Mcohtrnd18
Mcohtrnd20
Mcohtrnd22
Mcohtrnd24
Mcohtrnd26
Mcohtrnd28
Mcohtrnd30

!Names of net linear trends for wommen - see below for specifications
Fcohtrnd18
Fcohtrnd20
Fcohtrnd22
Fcohtrnd24
Fcohtrnd26
Fcohtrnd28
Fcohtrnd30
);
```

```
!Age specific specifications of net linear trends for men
Mcohtrnd18 = ((m5)+(m6-m5)+(m7-m6)+(m8-m7)+
              (m9-m8)+(m10-m9))/6;
Mcohtrnd20 = ((m11)+(m12-m11)+(m13-m12)+(m14-m13)+
              (m15-m14)+(m16-m15))/6;
Mcohtrnd22 = ((m17)+(m18-m17)+(m19-m18)+(m20-m19)+
              (m21-m20)+(m22-m21))/6;
Mcohtrnd24 = ((m23)+(m24-m23)+(m25-m24)+(m26-m25)+
              (m27-m26)+(m28-m27))/6;
Mcohtrnd26 = ((m29)+(m30-m29)+(m31-m30)+(m32-m31)+
              (m33-m32)+(m34-m33))/6;
Mcohtrnd28 = ((m35)+(m36-m35)+(m37-m36)+(m38-m37)+
              (m39-m38)+(m40-m39))/6;
Mcohtrnd28 = ((m41)+(m42-m41)+(m43-m42)+(m44-m43)+
              (m45-m44)+(m46-m45))/6;

!Age specific specifications of net linear trends for women
Fcohtrnd18 = ((f5)+(f6-f5)+(f7-f6)+(f8-f7)+
              (f9-f8)+(f10-f9))/6;
Fcohtrnd20 = ((f11)+(f12-f11)+(f13-f12)+(f14-f13)+
              (f15-f14)+(f16-f15))/6;
Fcohtrnd22 = ((f17)+(f18-f17)+(f19-f18)+(f20-f19)+
              (f21-f20)+(f22-f21))/6;
Fcohtrnd24 = ((f23)+(f24-f23)+(f25-f24)+(f26-f25)+
              (f27-f26)+(f28-f27))/6;
Fcohtrnd26 = ((f29)+(f30-f29)+(f31-f30)+(f32-f31)+
              (f33-f32)+(f34-f33))/6;
Fcohtrnd28 = ((f35)+(f36-f35)+(f37-f36)+(f38-f37)+
              (f39-f38)+(f40-f39))/6;
Fcohtrnd28 = ((f41)+(f42-f41)+(f43-f42)+(f44-f43)+
              (f45-f44)+(f46-f45))/6;




OUTPUT: Standardized samptat;
```
