# Supplementary material for: Age 18–30 Trajectories of Beverage‐Specific Binge Drinking Prevalence Over 30 Years for Men and Women in the United States
Source: Alcohol Clin Exp Res (Hoboken). 2026 Oct 15;50(10):e70420. doi: 10.1111/acer.70420 (PMC13673115; doi:10.1111/acer.70420)
Supplement: Supplementary file 2 [file ACER-50-0-s002.pdf]

```
TITLE:
   SUPPLEMENTAL MATERIALS
   EXAMPLE SYNTAX FOR AIM 1 - Age main effects

   Piece-wise latent growth model of binge drinking prevalence

   Biological sex included as grouping variable

   Model constraint command used to estimate gender differences

DATA:
    FILE IS [FILE NAME HERE]'
    FORMAT IS [FORMAT STATEMENT HERE];

  VARIABLE:
    NAMES ARE

     MTFID
     cPAWab !combination sample/attrition weight
     binge18 binge20 binge22 binge24 binge26 binge28 binge30 !Binge drinking indicators
     sex; !Biological sex measure



     USEVAR binge18 binge20 binge22 binge24 binge26 binge28 binge30;

     GROUPING is sex (0 = male 1 = female);


     MISSING ARE .;

     weight cPAWab;


ANALYSIS:
type = Meanstructure;
estimator = MLR;
iterations = 100000;
```

```
MODEL:

 !!! Specification of growth factors!!!
 Ibinge by binge18@1 binge20@1 binge22@1 binge24@1 binge26@1 binge28@1 binge30@1;
 L1binge by binge18@0 binge20@1 binge22@2 binge24@2 binge26@2 binge28@2 binge30@2;
 L2binge by binge18@0 binge20@0 binge22@0 binge24@1 binge26@2 binge28@2 binge30@2;
 L3binge by binge18@0 binge20@0 binge22@0 binge24@0 binge26@0 binge28@1 binge30@2;

 [Ibinge];
 [L1binge];
 [L2binge];
 [L3binge];

 Ibinge;
 L1binge;
 L2binge;
 L3binge;


 !specifying Covariances among growth factors
 Ibinge with L1binge L2binge L3binge;
 L1binge with L2binge L3binge;
 L2binge with L3binge;

 !Fixing means of growth factor indicators to zero
 [binge18@0]; [binge20@0]; [binge22@0];
 [binge24@0]; [binge26@0]; [binge28@0]; [binge30@0];

  !Specifying the variances of the growth factor indicators
  binge18; binge20; binge22;
  binge24; binge26; binge28; binge30;

Model Male:

 !!! Specification of growth factors!!!
```

```
 Ibinge by binge18@1 binge20@1 binge22@1 binge24@1 binge26@1 binge28@1 binge30@1;
 L1binge by binge18@0 binge20@1 binge22@2 binge24@2 binge26@2 binge28@2 binge30@2;
 L2binge by binge18@0 binge20@0 binge22@0 binge24@1 binge26@2 binge28@2 binge30@2;
 L3binge by binge18@0 binge20@0 binge22@0 binge24@0 binge26@0 binge28@1 binge30@2;

 [Ibinge];
 [L1binge];
 [L2binge];
 [L3binge];

 Ibinge;
 L1binge;
 L2binge;
 L3binge;


 !specifying Covariances among growth factors
 Ibinge with L1binge L2binge L3binge;
 L1binge with L2binge L3binge;
 L2binge with L3binge;

 !Fixing means of growth factor indicators to zero
 [binge18@0]; [binge20@0]; [binge22@0];
 [binge24@0]; [binge26@0]; [binge28@0]; [binge30@0];

  !Specifying the variances of the growth factor indicators
  binge18; binge20; binge22;
  binge24; binge26; binge28; binge30;



Model Female:

!!! Specification of growth factors!!!
 Ibinge by binge18@1 binge20@1 binge22@1 binge24@1 binge26@1 binge28@1 binge30@1;
 L1binge by binge18@0 binge20@1 binge22@2 binge24@2 binge26@2 binge28@2 binge30@2;
 L2binge by binge18@0 binge20@0 binge22@0 binge24@1 binge26@2 binge28@2 binge30@2;
 L3binge by binge18@0 binge20@0 binge22@0 binge24@0 binge26@0 binge28@1 binge30@2;
```

```
 [Ibinge];
 [L1binge];
 [L2binge];
 [L3binge];

 Ibinge;
 L1binge;
 L2binge;
 L3binge;


 !specifying Covariances among growth factors
 Ibinge with L1binge L2binge L3binge;
 L1binge with L2binge L3binge;
 L2binge with L3binge;

 !Fixing means of growth factor indicators to zero
 [binge18@0]; [binge20@0]; [binge22@0];
 [binge24@0]; [binge26@0]; [binge28@0]; [binge30@0];

  !Specifying the variances of the growth factor indicators
  binge18; binge20; binge22;
  binge24; binge26; binge28; binge30;


OUTPUT: standardized sampstat;
```
