# Supplementary material for: Age 18–30 Trajectories of Beverage‐Specific Binge Drinking Prevalence Over 30 Years for Men and Women in the United States
Source: Alcohol Clin Exp Res (Hoboken). 2026 Oct 15;50(10):e70420. doi: 10.1111/acer.70420 (PMC13673115; doi:10.1111/acer.70420)
Supplement: Supplementary file 3 [file ACER-50-0-s003.pdf]

Supplementary Table 1.Model fit indices and corresponding Santora-Bentler corrected chi-square difference tests

Model	Model fit						Chi-square difference tests			
Model	χ2	df	# of freely estimate parameters	CFI	RMSEA	Scaling correction factor	Models compared	Δχ2	Δdf	*p* -value
Age main effects (Aim 1)										
1. Overall binge drinking	31.376	28	42	1.000	.004	1.8032	--	--	--	--
2. Beer binge drinking	21.856	28	42	1.000	.000	2.0422	--	--	--	--
3. Wine binge drinking	47.137	28	42	.984	.010	3.5312	--	--	--	--
4. Liquor binge drinking	30.072	28	42	.999	.003	2.2036	--	--	--	--
Cohort main effects (Aim 2)										
5. Overall binge drinking, cohort group effects fixed across age	314.357	100	108	.989	.017	1.7891	--	--	--	--
6. Beer binge drinking, cohort group effects fixed across age	264.216	100	108	.991	.015	1.8271	--	--	--	--
7. Wine binge drinking, cohort group effects fixed across age	355.946	100	108	.976	.019	1.9642	--	--	--	--
8. Liquor binge drinking, cohort group effects fixed across age	211.241	100	108	.992	.012	1.8767	--	--	--	--
Cohort by age interactions (Aim 3)										
9. Overall binge, female cohort group effects free across age	176.514	64	144	.994	.016	1.8809	9 vs 5	141.713	36	<.001
10. Beer binge, female cohort group effects free across age	145.790	64	144	.996	.013	1.9822	10 vs 6	124.899	36	<.001
11.Wine binge, female cohort group effects free across age	146.882	64	144	.992	.013	2.1715	11 vs 7	238.267	36	<.001
12.Liqour binge, female cohort group effects free across age	109.930	64	144	.997	.010	1.9727	12 vs 8	105.260	36	<.001
13. Overall binge, male cohort group effects free across age	165.129	64	144	.995	.015	1.7171	13 vs 5	145.466	36	<.001
14. Beer binge, male cohort group effects free across age	132.801	64	144	.996	.012	1.7600	14 vs 6	127.939	36	<.001
15. Wine binge, male cohort group effects free across age	236.052	64	144	.984	.019	2.0685	15 vs 7	118.551	36	<.001
16. Liquor binge, male cohort group effects free across age	121.525	64	144	.996	.011	1.8587	16 vs 8	89.358	36	<.001

Notes. df = degrees of freedom. CFI = Comparative Fit Index. RMSEA = Room Mean Square of Approximation.

Supplementary Table 2. Age-varying effects of cohort group on binge drinking prevalence among women, by beverage type

Cohort group	Age 18	Age 20	Age 22	Age 24	Age 26	Age 28	Age 30
**Overall binge drinking**							
Intercept[1]	.312 (.287, .337)[a]	.314 (.292, .336)[a]	.316 (.291, .341)[a]	.270 (.248, .292)[a]	.223 (.199, .247)[a]	.184 (.164, .204)[a]	.145 (.121, .169)[a]
1993-1997	-.006 (-.041 .029)	-.008 (-.041, .025)	-.033 (-.068, .002)	-.016 (-.049, .017)	-.014 (-.047, .019)	-.009 (-.040, .022)	.009 (-.024, .042)
1998-2002	-.060 (-.095, -.025)[a]	-.045 (-.078, -.012)[a]	-.032 (-.069, .005)	-.033 (-.066, .000)	-.019 (-.054, .016)	-.010 (-.043, .023)	.001 (-.034, .036)
2003-2007	-.097 (-.132, -.062)[a]	-.064 (-.099, -.029)[a]	-.024 (-.063, .015)	-.009 (-.046, .028)	.032 (-.007, .071)	.038 (.001, .075)[a]	.053 (.016, .090)[a]
2008-2012	-.053 (-.092, -.014)[a]	-.027 (-.066, .012)	.036 (-.007, .079)	.071 (.030, .112)[a]	.087 (.044, .130)[a]	.097 (.056, .138)[a]	.098 (.055, .141)[a]
2013-2017	-.104 (-.143, -.065)[a]	-.072 (-.113, -.031)[a]	.054 (.009, .099)[a]	.052 (.009, .095)[a]	.059 (.014, .104)[a]	.105 (.060, .150)[a]	.078 (.035, .121)[a]
2018-2020	-.108 (-.153, -.063)[a]	-.064 (-.111, -.017)[a]	.055 (.000, .110)	.059 (.004, .114)[a]	.092 (.037, .147)[a]	.094 (.039, .149)[a]	.090 (.037, .143)[a]
Net-linear trend	-.018 (-.026, -.010)[a]	-.011 (-.021, -.001)[a]	.009 (-.001, .019)	.010 (.000, .020)	.015 (.003, .027)[a]	.016 (.006, .026)[a]	.015 (.005, .025)[a]
**Beer binge drinking**							
Intercept[1]	.249 (.225, .273)[a]	.227 (.207, .247)[a]	.206 (.182, .230)[a]	.176 (.158, .194)[a]	.145 (.123, .167)[a]	.123 (.105, .141)[a]	.101 (.081, .121)[a]
1993-1997	.025 (-.008, .058)	.027 (-.004, .058)	.023 (-.010, .056)	.020 (-.009, .049)	.026 (-.005, .057)	.010 (-.017, .037)	.023 (-.006, .052)
1998-2002	-.042 (-.075, -.009)[a]	-.025 (-.056, .006)	.016 (-.017, .049)	-.007 (-.038, .024)	-.010 (-.041, .021)	.005 (-.024, .034)	.014 (-.017, .045)
2003-2007	-.075 (-.110, -.040)[a]	-.041 (-.074, -.008)[a]	.007 (-.028, .042)	.013 (-.020, .046)	.036 (.001, .071)[a]	.043 (.008, .078)[a]	.034 (.001, .067)[a]
2008-2012	-.053 (-.090, -.016)[a]	-.018 (-.053, .017)	.038 (-.001, .077)	.073 (.036, .110)[a]	.067 (.028, .106)[a]	.046 (.011, .081)[a]	.052 (.017, .087)[a]
2013-2017	-.119 (-.156, -.082)[a]	-.076 (-.111, -.041)[a]	.018 (-.021, .057)	.011 (-.024, .046)	.038 (-.005, .081)	.073 (.030, .116)[a]	.055 (.016, .094)[a]
2018-2020	-.114 (-.157, -.071)[a]	-.080 (-.123, -.037)[a]	.019 (-.028, .066)	.061 (.012, .110)[a]	.055 (.006, .104)[a]	.028 (-.017, .073)	.031 (-.014, .076)
Net-linear trend	-.019 (-.027, -.011)[a]	-.013 (-.021, -.005)[a]	.003 (-.005, .011)	.010 (.000, .020)	.009 (.000, .018)	.005 (-.003, .013)	.005 (-.003, .013)

Cohort group	Wine binge drinking						
	Age 18	Age 20	Age 22	Age 24	Age 26	Age 28	Age 30
Intercept[1]	.109<br>(.093, .125)[a]	.114<br>(.102, .126)[a]	.119<br>(.103, .135)[a]	.110<br>(.098, .122)[a]	.102<br>(.086, 118)[a]	.078<br>(.066, .090)[a]	.053<br>(.039, .067)[a]
1993-1997	.017<br>(-.007 .041)	-.009<br>(-.031, .013)	-.027<br>(-.049, -.005)a	-.034<br>(-.054, -.014)a	-.032<br>(-.054, -.010)a	-.024<br>(-.044, -.004)a	-.005<br>(-.023, .013)
1998-2002	-.033<br>(-.055, -.011)[a]	-.057<br>(-.075, -.039)[a]	-.069<br>(-.089, -.049)a	-.056<br>(-.074, -.038)a	-.042<br>(-.064, -.020)a	-.026<br>(-.044, -.008)a	.003<br>(-.017, .023)
2003-2007	-.057<br>(-.079, -.035)[a]	-.076<br>(-.094, -.058)[a]	-.065<br>(-.087, -.043)a	-.050<br>(-.070, -.030)a	-.023<br>(-.047, .001)	.005<br>(-.019, .029)	.015<br>(-.007, .037)
2008-2012	-.055<br>(-.077, -.033)[a]	-.053<br>(-.075, -.031)[a]	-.023<br>(-.050, .004)	-.035<br>(-.057, -.013)a	.015<br>(-.014, .044)	.023<br>(-.002, .048)	.053<br>(.026, .080)a
2013-2017	-.064<br>(-.086, -.042)[a]	-.059<br>(-.081, -.037)[a]	-.022<br>(-.047, .003)	-.018<br>(-.042, .006)	.015<br>(-.014, .044)	.056<br>(.025, .087)a	.071<br>(.038, .104)a
2018-2020	-.057<br>(-.082, -.032)[a]	-.042<br>(-.069, -.015)[a]	.022<br>(-.015, .059)	.015<br>(-.018, .048)	.052<br>(.013, .091)a	.047<br>(.008, .086)a	.087<br>(.048, .126)a
Net-linear trend	-.010<br>(-.014, -.006)[a]	-.007<br>(-.011, -.003)[a]	.004<br>(.000, .008)	.003<br>(-.001, .007)	.009<br>(.005, .013)[a]	.008<br>(.004, .012)[a]	.015<br>(.011, .019)[a]

Cohort group	Liquor binge drinking						
	Age 18	Age 20	Age 22	Age 24	Age 26	Age 28	Age 30
Intercept[1]	.182<br>(.160, .204)[a]	.190<br>(.174, .206)[a]	.197<br>(.175, .219)[a]	.166<br>(.150, .182)[a]	.135<br>(.115, .155)[a]	.100<br>(.086, .114)[a]	.065<br>(.049, .081)[a]
1993-1997	.006<br>(-.023, .035)	.004<br>(-.023, .031)	-.008<br>(-.037, .021)	-.017<br>(-.042, .008)	-.036<br>(-.061, -.011)[a]	-.031<br>(-.053, -.009)[a]	-.011<br>(-.035, .013)
1998-2002	-.016<br>(-.045, .013)	-.032<br>(-.059, -.005)[a]	-.046<br>(-.075, -.017)[a]	-.042<br>(-.067, -.017)[a]	-.056<br>(-.081, -.031)[a]	-.011<br>(-.035, .013)	-.002<br>(-.026, .022)
2003-2007	-.017<br>(-.048, .014)	-.036<br>(-.065, -.007)[a]	-.035<br>(-.066, -.004)[a]	-.016<br>(-.045, .013)	.003<br>(-.030, .036)	.088<br>(.059, .117)[a]	.030<br>(.001, .059)[a]
2008-2012	.005<br>(-.030, .040)	.015<br>(-.018, .048)	.039<br>(.002, .076)[a]	.052<br>(.017, .087)[a]	.087<br>(.048, .126)[a]	.069<br>(.034, .104)[a]	.106<br>(.071, .141)[a]
2013-2017	.002<br>(-.033, .037)	.025<br>(-.012, .062)	.086<br>(.045, .127)[a]	.091<br>(.052, 130)[a]	.046<br>(.009, .083)[a]	.116<br>(.075, .157)[a]	.084<br>(.043, .125)[a]
2018-2020	.003<br>(-.038, .044)	.021<br>(-.022, .064)	.114<br>(.063, .165)[a]	.104<br>(.055, .153)[a]	.075<br>(.024, .126)[a]	.094<br>(.047, .141)[a]	.111<br>(.064, .158)[a]
Net-linear trend	.001<br>(-.007, .009)	.004<br>(-.004, .012)	.019<br>(.011, .027)[a]	.017<br>(.009, .025)[a]	.012<br>(.004, .020)[a]	.016<br>(.008, .024)[a]	.019<br>(.011, .027)[a]

Notes. 95% confidence intervals are provided in parentheses. [1]Intercepts are mean estimates of binge drinking for refererence group (i.e., 1988-1992 cohort group). [a]Statistically significant finding (95% confidence interval excludes 0).

Supplementary Table 3. Age-varying effects of cohort group on binge drinking prevalence among men, by beverage type

	Overall binge drinking						
Cohort group	Age 18	Age 20	Age 22	Age 24	Age 26	Age 28	Age 30
Intercept[1]	.475 (.446, .504)[a]	.507 (.482, .532)[a]	.538 .509, .567)[a]	.489 .464, .514)[a]	.439 .410, .468)[a]	.407 (.380, .434)[a]	.375 (.344, .406)[a]
1993-1997	.002 (-.039, .043)	.000 (-.039, .039)	-.026 (-.067, .015)	-.008 (-.047, .031)	-.007 (-.048, .034)	-.001 (-.042, .040)	.016 (-.027, .059)
1998-2002	-.073 (-.114, -.032)[a]	-.058 (-.099, -.017)[a]	-.044 (-.087, -.001)[a]	-.045 (-.086, -.004)[a]	-.032 (-.075, .011)	-.023 (-.066, .020)	-.011 (-.056, .034)
2003-2007	-.124 (-.167, -.081)[a]	-.092 (-.135, -.049)[a]	-.052 (-.099, -.005)[a]	-.037 (-.082, .008)	.005 (-.042, .052)	.010 (-.035, .055)	.025 (-.022, .072)
2008-2012	-.111 (-.156, -.066)[a]	-.085 (-.132, -.038)[a]	-.022 (-.073, .029)	.013 (-.036, .062)	.029 (-.022, .080)	.039 (-.010, .088)	.040 (-.011, .091)
2013-2017	-.144 (-.193, -.095)[a]	-.112 (-.161, -.063)[a]	.014 (-.039, .067)	.012 (-.041, .065)	.018 (-.035, .071)	.065 (.010, .120)[a]	.038 (-.019, .095)
2018-2020	-.236 (-.289, -.183)[a]	-.193 (-.250, -.136)[a]	-.073 (-.136, -.010)[a]	-.069 (-.130, -.008)[a]	-.036 (-.101, .029)	-.035 (-.100, .030)	-.038 (-.101, .025)
Net-linear trend	-.039 (-.047, -.031)[a]	-.032 (-.042, -.022)[a]	-.012 (-.022, -.002)[a]	-.012 (-.022, -.002)[a]	-.006 (-.016, .004)	-.006 (-.016, .004)	-.006 (-.016, .004)
	Beer binge drinking						
Cohort group	Age 18	Age 20	Age 22	Age 24	Age 26	Age 28	Age 30
Intercept[1]	.455 (.426, .484)[a]	.475 (.450, .500)[a]	.494 (.465, .523)[a]	.456 (.431, .481)[a]	.417 (.388, .446)[a]	.394 (.367, .421)[a]	.371 (.342, .400)[a]
1993-1997	.017 (-.024, .058)	.018 (-.021, .057)	.014 (-.027, .055)	.011 (-.028, .050)	.017 (-.024, .058)	.002 (-.037, .041)	.014 (-.027, .055)
1998-2002	-.066 (-.107, -.025)[a]	-.049 (-.090, -.008)[a]	-.008 (-.051, .035)	-.031 (-.072, .010)	-.034 (-.075, .007)	-.019 (-.060, .022)	-.010 (-.053, .033)
2003-2007	-.110 (-.153, -.067)[a]	-.076 (-.117, -.035)[a]	-.027 (-.072, .018)	-.021 (-.064, .022)	.001 (-.044, .046)	.009 (-.034, .052)	.000 (-.045, .045)
2008-2012	-.104 (-.151, -.057)[a]	-.069 (-.114, -.024)[a]	-.013 (-.062, .036)	.022 (-.025, .069)	.016 (-.033, .065)	-.006 (-.053, .041)	.001 (-.048, .050)
2013-2017	-.136 (-.183, -.089)[a]	-.093 (-.140, -.046)[a]	.000 (-.051, .051)	-.006 (-.055, .043)	.020 (-.031, .071)	.056 (.003, .109)[a]	.038 (-.015, .091)
2018-2020	-.211 (-.264, -.158)[a]	-.177 (-.228, -.126)[a]	-.079 (-.138, -.020)[a]	-.036 (-.095, .023)	-.043 (-.104, .018)	-.070 (-.129, -.011)[a]	-.067 (-.126, -.008)[a]
Net-linear trend	-.035 (-.045, -.025)[a]	-.030 (-.038, -.022)[a]	-.013 (-.023, -.003)[a]	-.006 (-.016, .004)	-.007 (-.017, .003)	-.008 (-.018, .002)	-.008 (-.018, .002)

	Wine binge drinking						
Cohort group	Age 18	Age 20	Age 22	Age 24	Age 26	Age 28	Age 30
Intercept[1]	.118<br>(.100, .136)[a]	.120<br>(.106, .134)[a]	.121<br>(.103, .139)[a]	.113<br>(.099, .127)[a]	.105<br>(.087, .123)[a]	.080<br>(.068, .092)[a]	.055<br>(.039, .071)[a]
1993-1997	.020<br>(-.005, .045)	-.006<br>(-.028, .016)	-.024<br>(-.048, .000)	-.031<br>(-.053, -.009)[a]	-.029<br>(-.051, -.007)[a]	-.021<br>(-.041, -.001)[a]	-.002<br>(-.022, .018)
1998-2002	-.033<br>(-.055, -.011)a	-.057<br>(-.077, -.037)[a]	-.069<br>(-.091, -.047)[a]	-.056<br>(-.076, -.036)[a]	-.042<br>(-.066, -.018)[a]	-.026<br>(-.044, -.008)[a]	.003<br>(-.019, .025)
2003-2007	-.056<br>(-.080, -.032)a	-.075<br>(-.095, -.055)[a]	-.064<br>(-.088, -.040)[a]	-.049<br>(-.071, -.027)[a]	-.022<br>(-.047, .003)	.005<br>(-.019, .029)	.016<br>(-.009, .041)
2008-2012	-.056<br>(-.080, -.032)a	-.054<br>(-.076, -.032)[a]	-.024<br>(-.049, .001)	-.036<br>(-.060, -.012)[a]	.014<br>(-.017, .045)	.022<br>(-.005, .049)	.052<br>(.023, .081)[a]
2013-2017	-.078<br>(-.102, -.054)a	-.072<br>(-.094, -.050)[a]	-.035<br>(-.062, -.008)[a]	-.031<br>(-.056, -.006)[a]	.001<br>(-.028, .030)	.042<br>(.011, .073)[a]	.057<br>(.026, .088)[a]
2018-2020	-.095<br>(-.122, -.068)a	-.080<br>(-.104, -.056)[a]	-.016<br>(-.055, .023)	-.023<br>(-.056, .010)	.014<br>(-.025, .053)	.009<br>(-.030, .048)	.049<br>(.012, .086)[a]
Net-linear trend	-.016<br>(-.020, -.012)[a]	-.013<br>(-.017, -.009)[a]	-.003<br>(-.009, .003)	-.004<br>(-.010, .002)	.002<br>(-.004, .008)	.001<br>(-.005, .007)	.008<br>(.002, .014)[a]

	Liquor binge drinking						
Cohort group	Age 18	Age 20	Age 22	Age 24	Age 26	Age 28	Age 30
Intercept[1]	.246<br>(.222, .270)[a]	.258<br>(.238, .278)[a]	.270<br>(.246, .294)[a]	.240<br>(.220, .260)[a]	.209<br>(.185, .233)[a]	.181<br>(.161, .201)a	.152<br>(.128, .176)[a]
1993-1997	.001<br>(-.030, .032)	-.001<br>(-.032, .030)	-.013<br>(-.046, .020)	-.023<br>(-.054, .008)	-.041<br>(-.072, -.010)[a]	-.036<br>(-.063, -.009)[a]	-.016<br>(-.045, .013)
1998-2002	-.029<br>(-.062, .004)	-.044<br>(-.075, -.013)[a]	-.059<br>(-.092, -.026)[a]	-.055<br>(-.086, -.024)[a]	-.068<br>(-.099, -.037)[a]	-.023<br>(-.054, .008)	-.015<br>(-.046, .016)
2003-2007	-.020<br>(-.055, .015)	-.039<br>(-.072, -.006)[a]	-.038<br>(-.075, -.001)[a]	-.019<br>(-.054, .016)	.000<br>(-.037, .037)	.005<br>(-.030, .040)	.027<br>(-.008, .062)
2008-2012	-.016<br>(-.053, .021)	-.006<br>(-.043, .031)	.018<br>(-.023, .059)	.031<br>(-.008, .070)	.066<br>(.025, .107)[a]	.048<br>(.009, .087)[a]	.085<br>(.044, .126)[a]
2013-2017	.015<br>(-.026, .056)	.038<br>(-.003, .079)	.099<br>(.054, .144)[a]	.104<br>(.059, .149)[a]	.059<br>(.014, .104)[a]	.128<br>(.081, .175)[a]	.097<br>(.050, .144)[a]
2018-2020	-.031<br>(-.082, .020)	-.013<br>(-.064, .038)	.079<br>(.022, .136)[a]	.070<br>(.013, .127)[a]	.041<br>(-.014, .096)	.060<br>(.003, .117)[a]	.077<br>(.020, .134)[a]
Net-linear trend	-.005<br>(-.013, .003)	-.002<br>(-.010, .006)	.013<br>(.003, .023)[a]	.012<br>(.002, .022)[a]	.007<br>(-.003, .017)	.011<br>(.001, .021)[a]	.013<br>(.003, .023)[a]

Notes. 95% confidence intervals are provided in parentheses. [1]Intercepts are mean estimates of binge drinking for refererence group (i.e., 1988-1992 cohort group). [a]Statistically significant finding (95% confidence interval excludes 0).

Supplementary Table 4. The distribution of reported beverage-specific types of binge drinking among those who reported overall binge drinking by age

Type(s) of beverage-specific binge drinking reported[1]	Age 18 Rel. % (95% CI)	Age 20 Rel. % (95% CI)	Age 22 Rel. % (95% CI)	Age 24 Rel. % (95% CI)	Age 26 Rel. % (95% CI)	Age 28 Rel. % (95% CI)	Age 30 Rel. % (95% CI)
Beer only	33.1 (31.1, 35.1)	34.1 (32.3, 35.9)	36.1 (34.3, 37.9)	38.0 (36.0, 40.0)	39.3 (37.1, 41.5)	41.2 (38.8, 43.6)	42.8 (40.2, 45.4)
Wine only	1.7 (1.2, 2.2)	1.8 (1.3, 2.3)	1.6 (1.1, 2.1)	1.6 (1.1, 2.1)	3.2 (2.4, 4.0)	3.1 (2.3, 3.9)	3.8 (2.8, 4.8)
Liquor only	9.0 (7.8, 10.2)	10.5 (9.3, 11.7)	10.5 (9.3, 11.7)	10.9 (9.6, 12.2)	9.5 (8.2, 10.8)	9.2 (7.8, 10.6)	9.6 (8.0, 11.2)
Beer and wine	4.2 (3.4, 5.0)	3.2 (2.5, 3.9)	4.3 (3.6, 5.0)	4.4 (3.6, 5.2)	5.7 (4.7, 6.7)	5.9 (4.7, 7.1)	5.9 (4.8, 7.0)
Beer and liquor	30.2 (28.4, 32.0)	31.3 (29.5, 33.1)	28.6 (26.9, 30.3)	26.7 (24.9, 28.5)	22.3 (20.4, 24.2)	19.7 (19.5, 19.9)	19.5 (16.7), 22.3)
Wine and liquor	1.6 (1.2, 2.0)	2.1 (1.5, 2.7)	2.3 (1.7, 2.9)	2.4 (1.8, 3.0)	3.2 (2.4, 4.0)	2.3 (1.7, 2.9)	2.7 (1.8, 3.6)
Beer, wine, and liquor	11.9 (10.7, 13.1)	9.0 (7.9, 10.1)	9.2 (8.1, 10.3)	8.2 (7.1, 9.3)	8.5 (7.3, 9.7)	8.7 (7.3, 10.1)	7.6 (6.1, 9.1)
None	8.3 (7.1, 9.5)	8.1 (7.0, 9.2)	7.4 (6.4, 8.4)	7.8 (6.6, 9.0)	8.3 (7.0, 9.6)	10.0 (8.5, 11.5)	8.1 (6.6, 9.6)

Notes. 95% confidence intervals are provided in parentheses.
